# Supplementary material for: Can the Kuznetsov Model Replicate and Predict Cancer Growth in Humans?
Source: Bull Math Biol. 2022 Sep 29;84(11):130. doi: 10.1007/s11538-022-01075-7 (PMC9522842; doi:10.1007/s11538-022-01075-7)
Supplement: Supplementary file 1 — (pdf 0 KB) [file 11538_2022_1075_MOESM1_ESM.pdf]

Supplementary Material for: Can the Kuznetsov Model Replicate and Predict Cancer Growth in Humans?

Mohammad El Wajeh[1], Falco Jung[1], Dominik Bongartz[1], Chrysoula Dimitra Kappatou[2], Narmin Ghaffari Laleh[3], Alexander Mitsos[4,1,5,*], and Jakob Nikolas Kather[3,6,*]

[1] *Process Systems Engineering (AVT.SVT), RWTH Aachen University, 52074 Aachen, Germany*
[2] *Faculty of Engineering, Department of Computing, Imperial College London, London SW7 2AZ, UK*
[3] *Department of Medicine III, University Hospital RWTH Aachen, 52074 Aachen, Germany*
[4] *JARA-CSD, 52056 Aachen, Germany*
[5] *Energy Systems Engineering (IEK-10), Forschungszentrum Jülich, 52425 Jülich, Germany*
[6] *Medical Oncology, National Center for Tumor Diseases, University Hospital Heidelberg, 69120 Heidelberg, Germany*
[*] *Shared last authorship and corresponding authors: amitsos@alum.mit.edu, jkather@ukaachen.de*

Estimated values of the model parameters of the 111 patients from study 4 (after data pretreatment), shown per each arm in the study. The bottom and top of the boxes of the box plots are the 25th and 75th percentiles of the data, respectively. The distance between the bottom and top of each box is the interquartile range. The red line in the middle of each box is the data median. The outliers (red plus sign) are the values that are more than 1.5 times the interquartile range away from the bottom or top of the box. Ordinates: parameter values. Abscissas: non-dimensionalized model parameters. The values are scattered all over the bounds' ranges, but α values, which have a certain maximum. Moreover, the distribution densities of parameter values are very close to each other between the two arms.

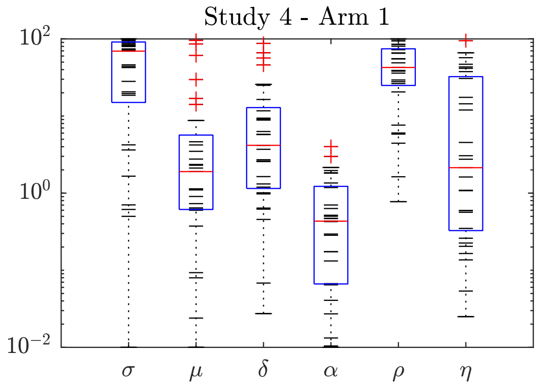

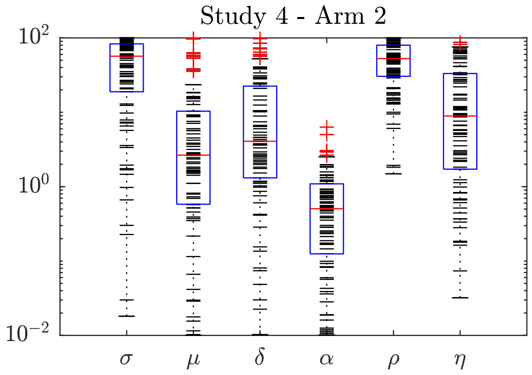

Model extrapolation results of the selected patients. The solid-line curves and the points represent model results and measured data, respectively. The last three data points are not used for parameters estimation. Ordinates: normalized number of tumor cells. Abscissas: normalized treatment time, negative values indicate time before the start of treatment. The model is capable of forecasting tumor dynamics qualitatively and sometimes quantitatively.

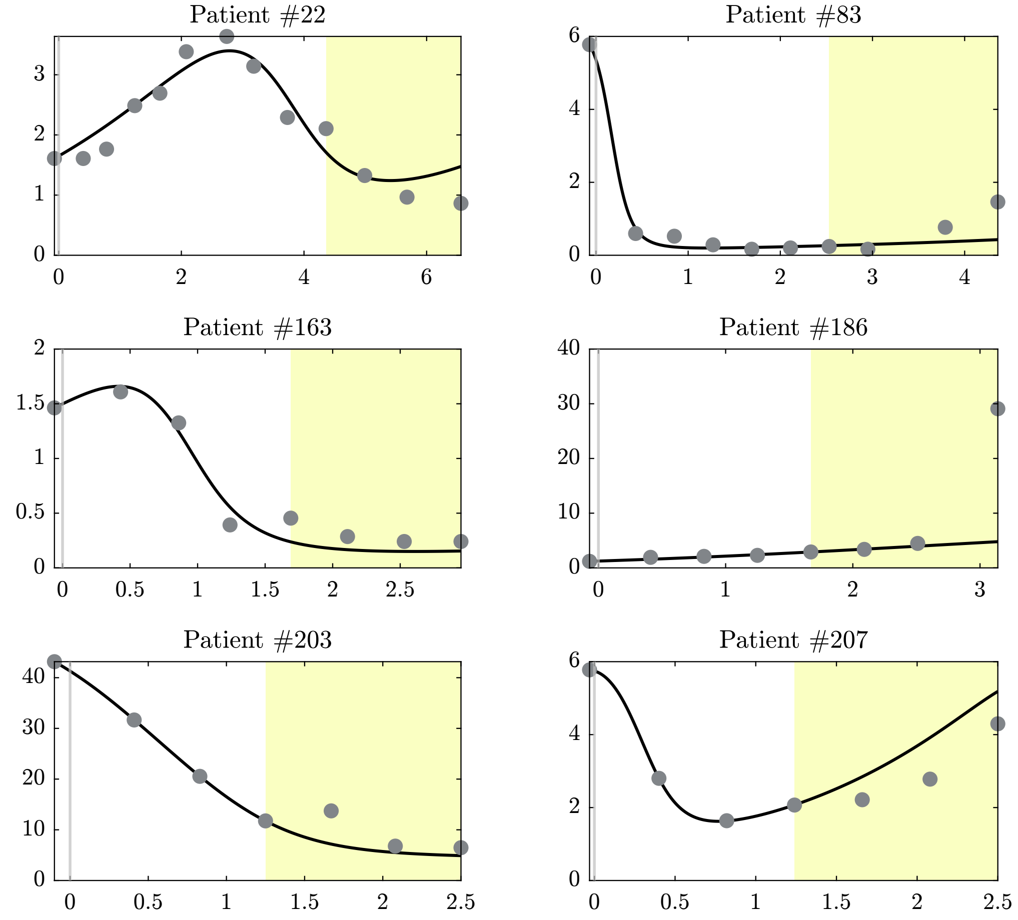

Data fitting results of TC number of all 210 patients. The solid (black) line shows model results, where all data points are used when estimating the parameters. The points represent the measured data. Ordinates: normalized number of tumor cells. Abscissas: normalized treatment time, where negative values indicate time before the start of treatment. The model can fit experimental data with different qualitative trends (e.g., up, down and "U"-curve).

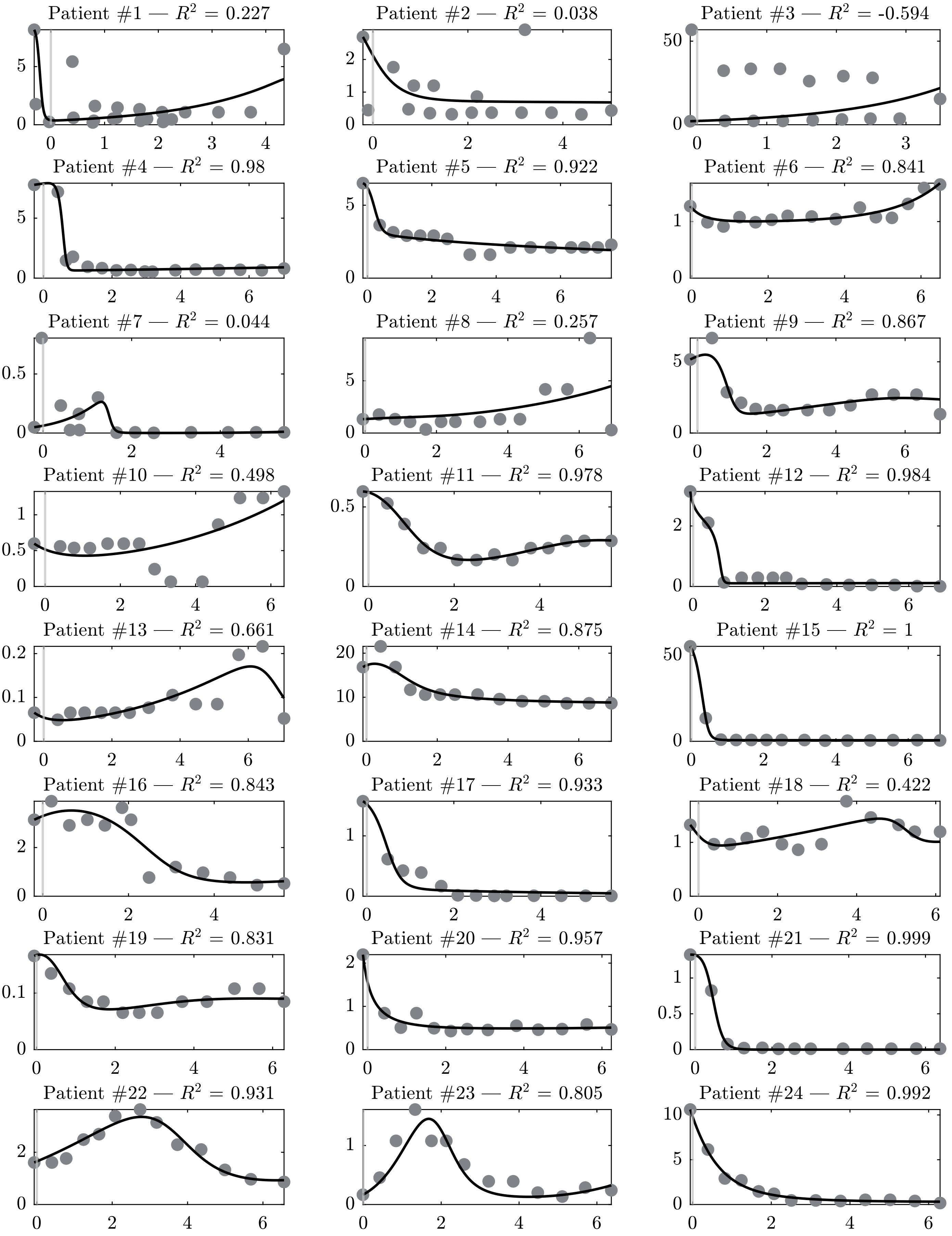
Patient #1 — R^2 = 0.227
Patient #2 — R^2 = 0.038
Patient #3 — R^2 = -0.594
Patient #4 — R^2 = 0.98
Patient #5 — R^2 = 0.922
Patient #6 — R^2 = 0.841
Patient #7 — R^2 = 0.044
Patient #8 — R^2 = 0.257
Patient #9 — R^2 = 0.867
Patient #10 — R^2 = 0.498
Patient #11 — R^2 = 0.978
Patient #12 — R^2 = 0.984
Patient #13 — R^2 = 0.661
Patient #14 — R^2 = 0.875
Patient #15 — R^2 = 1
Patient #16 — R^2 = 0.843
Patient #17 — R^2 = 0.933
Patient #18 — R^2 = 0.422
Patient #19 — R^2 = 0.831
Patient #20 — R^2 = 0.957
Patient #21 — R^2 = 0.999
Patient #22 — R^2 = 0.931
Patient #23 — R^2 = 0.805
Patient #24 — R^2 = 0.992

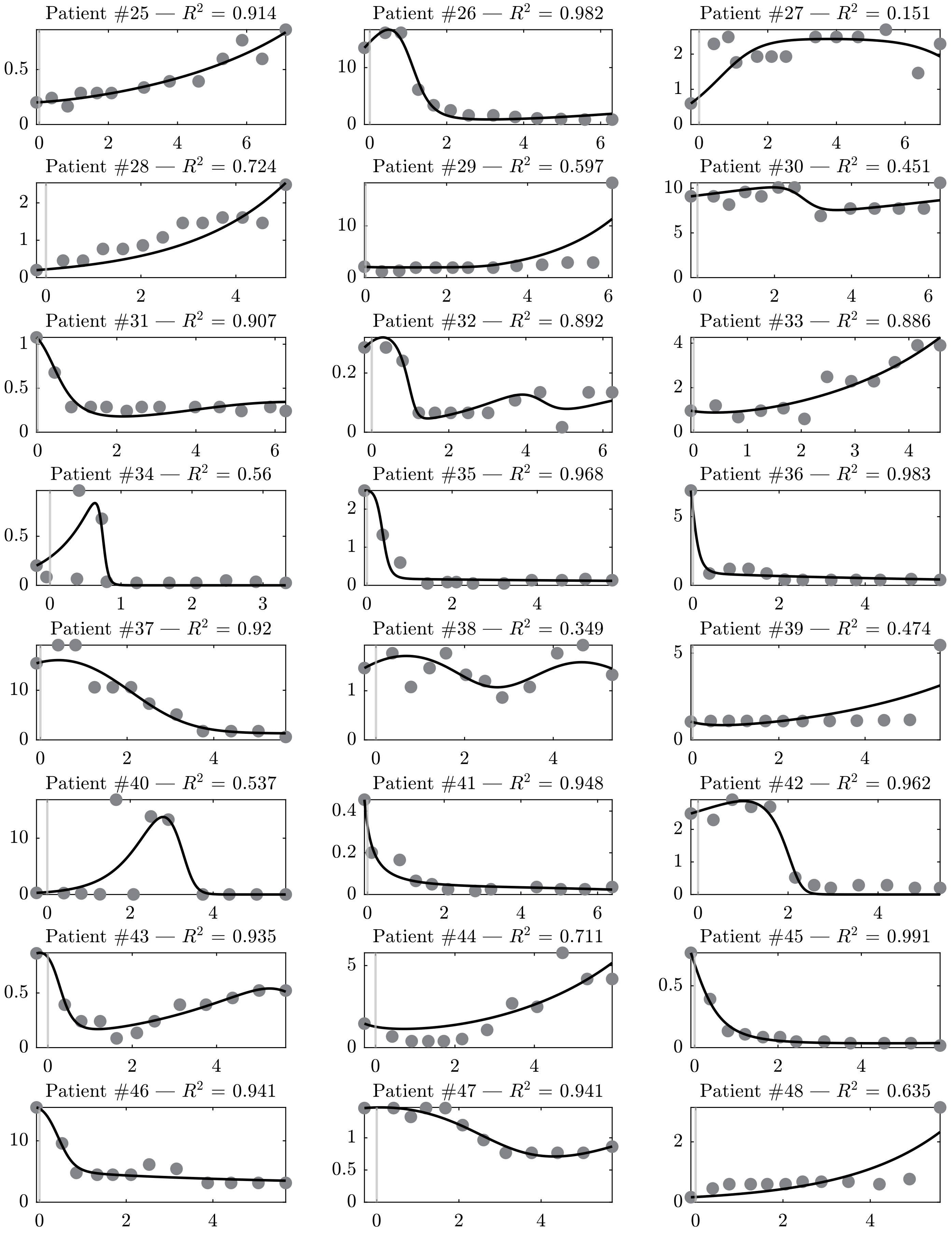
Patient #25 — R^2 = 0.914
Patient #26 — R^2 = 0.982
Patient #27 — R^2 = 0.151
Patient #28 — R^2 = 0.724
Patient #29 — R^2 = 0.597
Patient #30 — R^2 = 0.451
Patient #31 — R^2 = 0.907
Patient #32 — R^2 = 0.892
Patient #33 — R^2 = 0.886
Patient #34 — R^2 = 0.56
Patient #35 — R^2 = 0.968
Patient #36 — R^2 = 0.983
Patient #37 — R^2 = 0.92
Patient #38 — R^2 = 0.349
Patient #39 — R^2 = 0.474
Patient #40 — R^2 = 0.537
Patient #41 — R^2 = 0.948
Patient #42 — R^2 = 0.962
Patient #43 — R^2 = 0.935
Patient #44 — R^2 = 0.711
Patient #45 — R^2 = 0.991
Patient #46 — R^2 = 0.941
Patient #47 — R^2 = 0.941
Patient #48 — R^2 = 0.635

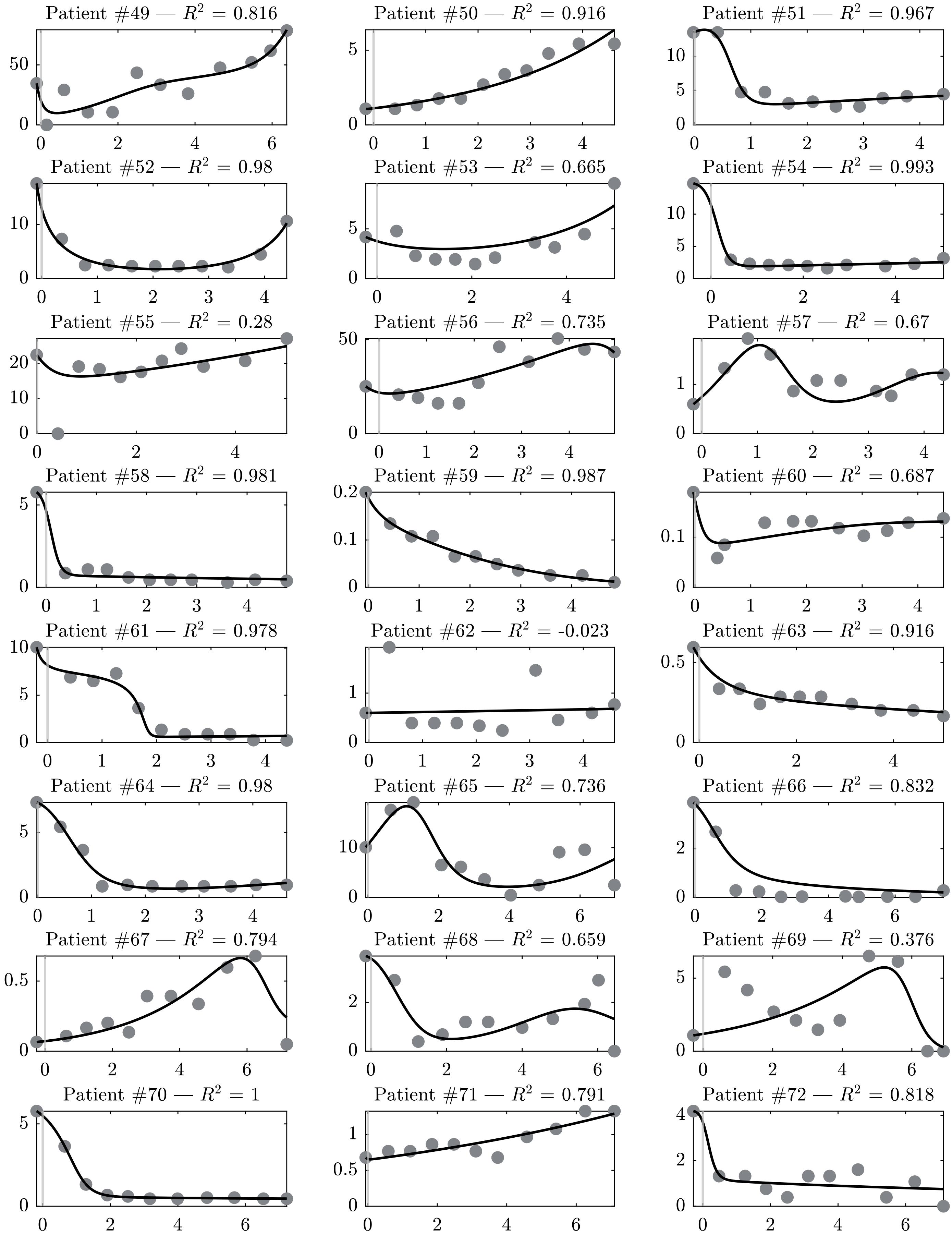
Patient #49 — R^2 = 0.816
0 50
0 2 4 6
Patient #50 — R^2 = 0.916
0 5
0 1 2 3 4
Patient #51 — R^2 = 0.967
0 5 10
0 1 2 3 4
Patient #52 — R^2 = 0.98
0 10
0 1 2 3 4
Patient #53 — R^2 = 0.665
0 5
0 2 4
Patient #54 — R^2 = 0.993
0 5 10
0 2 4
Patient #55 — R^2 = 0.28
0 10 20
0 2 4
Patient #56 — R^2 = 0.735
0 50
0 1 2 3 4
Patient #57 — R^2 = 0.67
0 1
0 1 2 3 4
Patient #58 — R^2 = 0.981
0 5
0 1 2 3 4
Patient #59 — R^2 = 0.987
0 0.1 0.2
0 1 2 3 4
Patient #60 — R^2 = 0.687
0 0.1
0 1 2 3 4
Patient #61 — R^2 = 0.978
0 5 10
0 1 2 3 4
Patient #62 — R^2 = -0.023
0 1
0 1 2 3 4
Patient #63 — R^2 = 0.916
0 0.5
0 2 4
Patient #64 — R^2 = 0.98
0 5
0 1 2 3 4
Patient #65 — R^2 = 0.736
0 10
0 2 4 6
Patient #66 — R^2 = 0.832
0 2
0 2 4 6
Patient #67 — R^2 = 0.794
0 0.5
0 2 4 6
Patient #68 — R^2 = 0.659
0 2
0 2 4 6
Patient #69 — R^2 = 0.376
0 5
0 2 4 6
Patient #70 — R^2 = 1
0 5
0 2 4 6
Patient #71 — R^2 = 0.791
0 0.5 1
0 2 4 6
Patient #72 — R^2 = 0.818
0 2 4
0 2 4 6

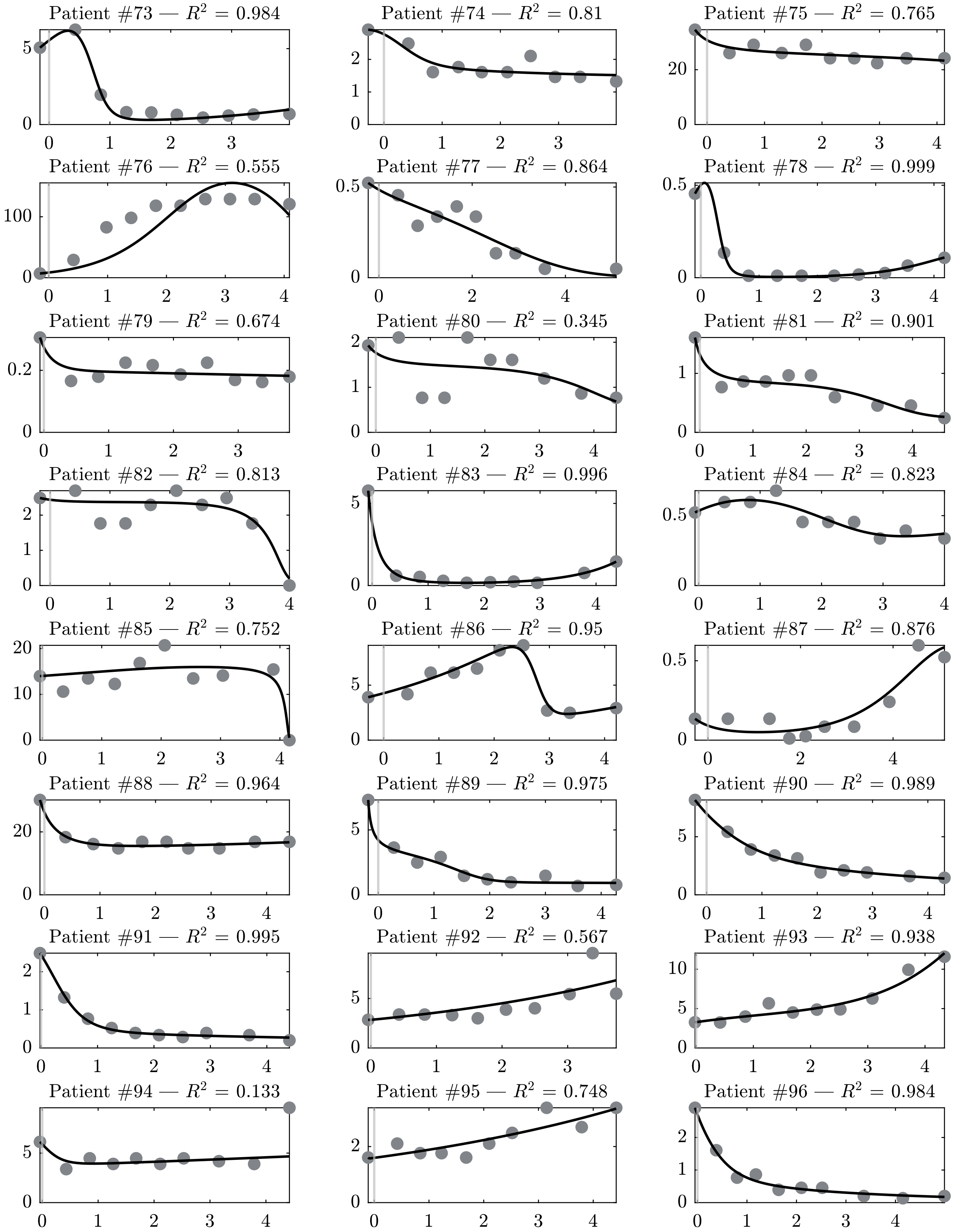
Patient #73 — R^2 = 0.984
Patient #74 — R^2 = 0.81
Patient #75 — R^2 = 0.765
Patient #76 — R^2 = 0.555
Patient #77 — R^2 = 0.864
Patient #78 — R^2 = 0.999
Patient #79 — R^2 = 0.674
Patient #80 — R^2 = 0.345
Patient #81 — R^2 = 0.901
Patient #82 — R^2 = 0.813
Patient #83 — R^2 = 0.996
Patient #84 — R^2 = 0.823
Patient #85 — R^2 = 0.752
Patient #86 — R^2 = 0.95
Patient #87 — R^2 = 0.876
Patient #88 — R^2 = 0.964
Patient #89 — R^2 = 0.975
Patient #90 — R^2 = 0.989
Patient #91 — R^2 = 0.995
Patient #92 — R^2 = 0.567
Patient #93 — R^2 = 0.938
Patient #94 — R^2 = 0.133
Patient #95 — R^2 = 0.748
Patient #96 — R^2 = 0.984

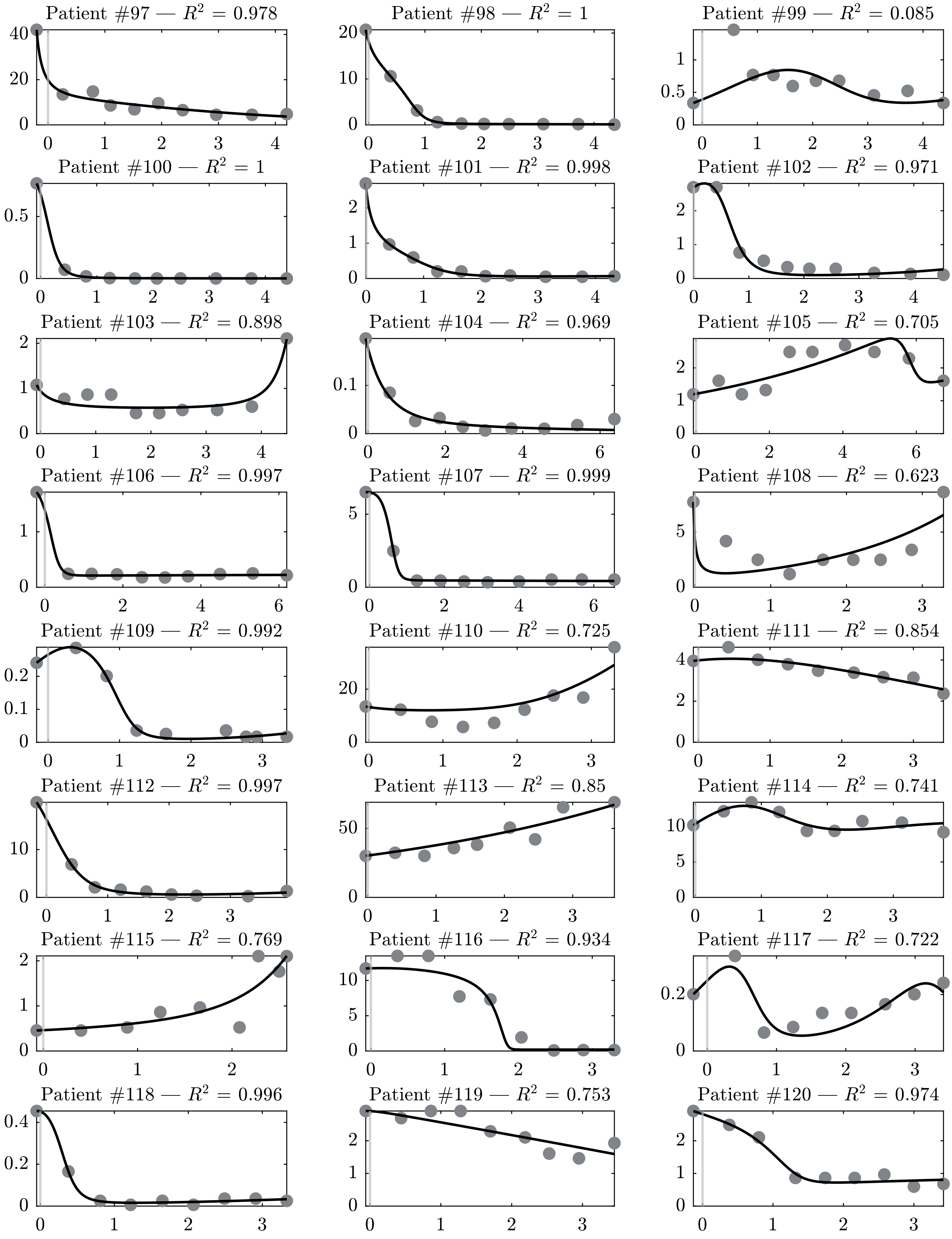

Patient #97 — R^2 = 0.978
Patient #98 — R^2 = 1
Patient #99 — R^2 = 0.085
Patient #100 — R^2 = 1
Patient #101 — R^2 = 0.998
Patient #102 — R^2 = 0.971
Patient #103 — R^2 = 0.898
Patient #104 — R^2 = 0.969
Patient #105 — R^2 = 0.705
Patient #106 — R^2 = 0.997
Patient #107 — R^2 = 0.999
Patient #108 — R^2 = 0.623
Patient #109 — R^2 = 0.992
Patient #110 — R^2 = 0.725
Patient #111 — R^2 = 0.854
Patient #112 — R^2 = 0.997
Patient #113 — R^2 = 0.85
Patient #114 — R^2 = 0.741
Patient #115 — R^2 = 0.769
Patient #116 — R^2 = 0.934
Patient #117 — R^2 = 0.722
Patient #118 — R^2 = 0.996
Patient #119 — R^2 = 0.753
Patient #120 — R^2 = 0.974

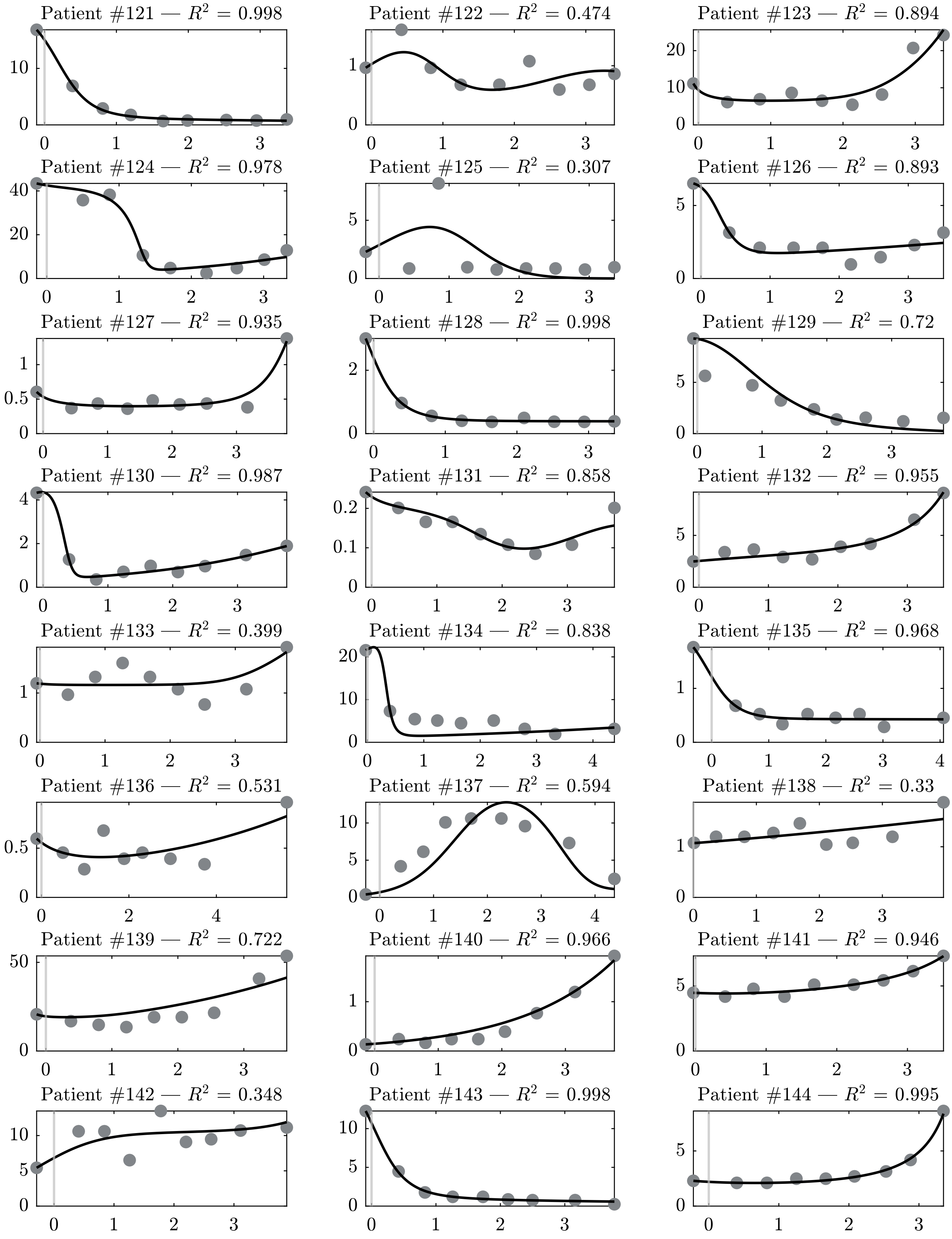
Patient #121 — R^2 = 0.998
Patient #122 — R^2 = 0.474
Patient #123 — R^2 = 0.894
Patient #124 — R^2 = 0.978
Patient #125 — R^2 = 0.307
Patient #126 — R^2 = 0.893
Patient #127 — R^2 = 0.935
Patient #128 — R^2 = 0.998
Patient #129 — R^2 = 0.72
Patient #130 — R^2 = 0.987
Patient #131 — R^2 = 0.858
Patient #132 — R^2 = 0.955
Patient #133 — R^2 = 0.399
Patient #134 — R^2 = 0.838
Patient #135 — R^2 = 0.968
Patient #136 — R^2 = 0.531
Patient #137 — R^2 = 0.594
Patient #138 — R^2 = 0.33
Patient #139 — R^2 = 0.722
Patient #140 — R^2 = 0.966
Patient #141 — R^2 = 0.946
Patient #142 — R^2 = 0.348
Patient #143 — R^2 = 0.998
Patient #144 — R^2 = 0.995

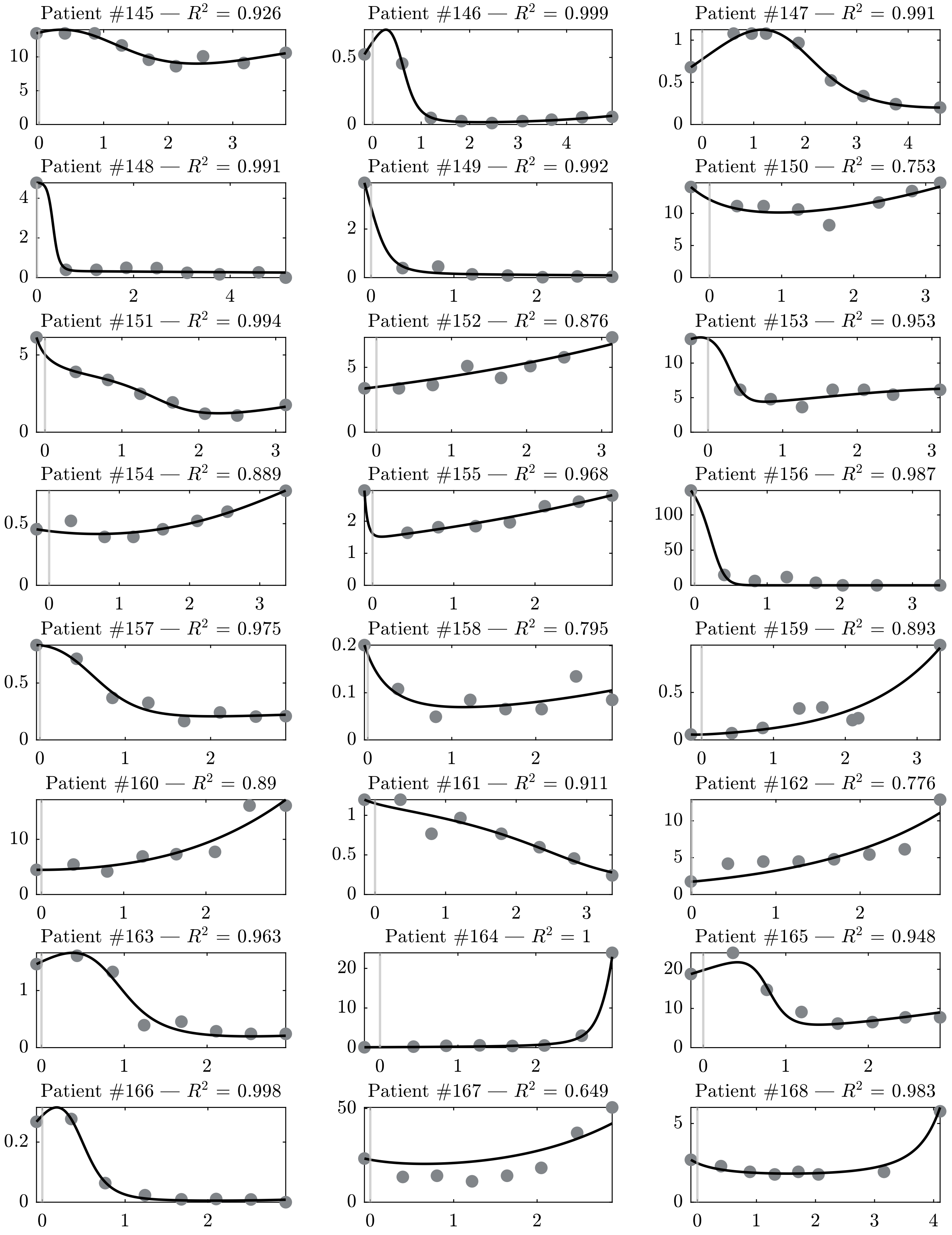
Patient #145 — R^2 = 0.926
Patient #146 — R^2 = 0.999
Patient #147 — R^2 = 0.991
Patient #148 — R^2 = 0.991
Patient #149 — R^2 = 0.992
Patient #150 — R^2 = 0.753
Patient #151 — R^2 = 0.994
Patient #152 — R^2 = 0.876
Patient #153 — R^2 = 0.953
Patient #154 — R^2 = 0.889
Patient #155 — R^2 = 0.968
Patient #156 — R^2 = 0.987
Patient #157 — R^2 = 0.975
Patient #158 — R^2 = 0.795
Patient #159 — R^2 = 0.893
Patient #160 — R^2 = 0.89
Patient #161 — R^2 = 0.911
Patient #162 — R^2 = 0.776
Patient #163 — R^2 = 0.963
Patient #164 — R^2 = 1
Patient #165 — R^2 = 0.948
Patient #166 — R^2 = 0.998
Patient #167 — R^2 = 0.649
Patient #168 — R^2 = 0.983

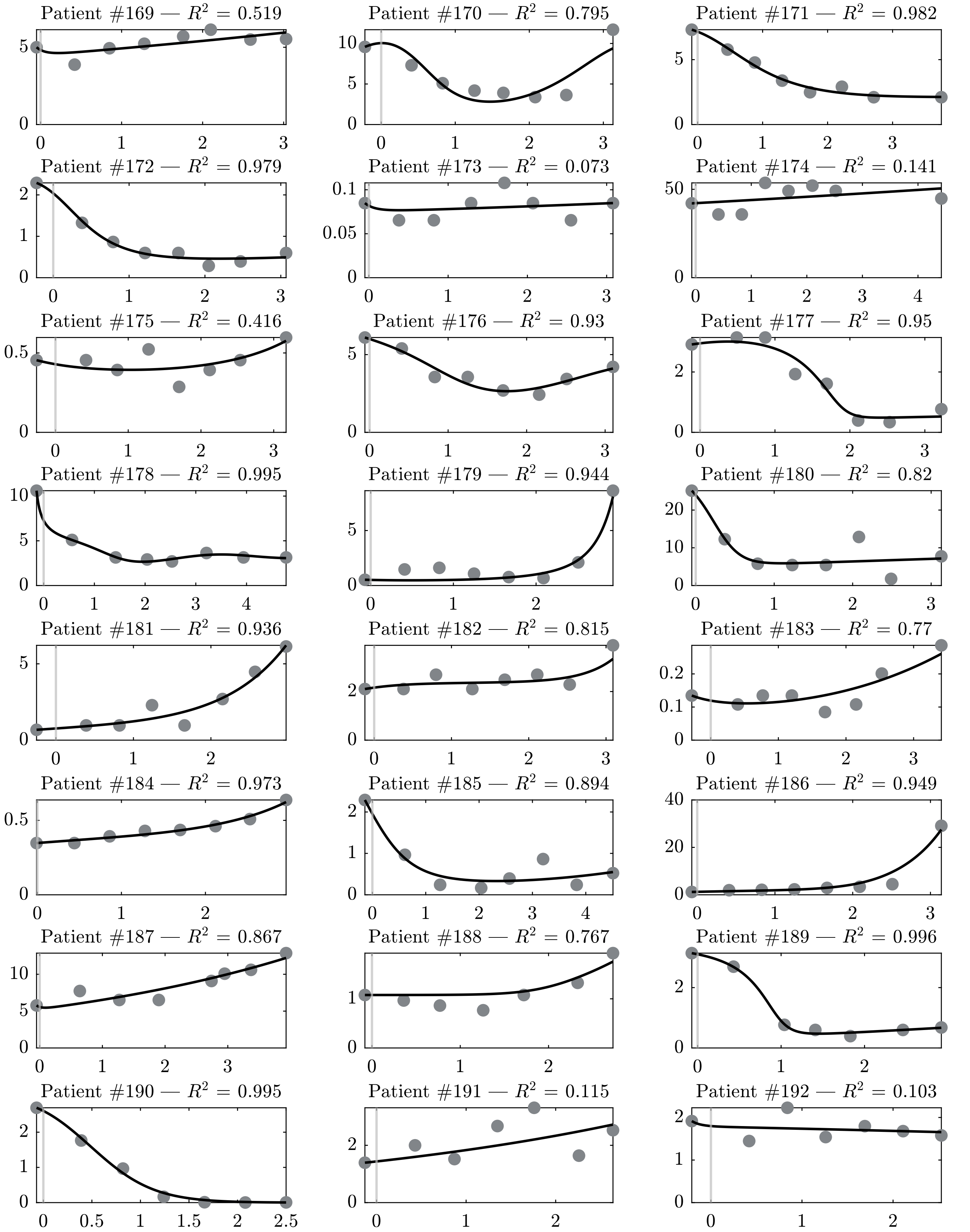
Patient #169 — R^2 = 0.519
Patient #170 — R^2 = 0.795
Patient #171 — R^2 = 0.982
Patient #172 — R^2 = 0.979
Patient #173 — R^2 = 0.073
Patient #174 — R^2 = 0.141
Patient #175 — R^2 = 0.416
Patient #176 — R^2 = 0.93
Patient #177 — R^2 = 0.95
Patient #178 — R^2 = 0.995
Patient #179 — R^2 = 0.944
Patient #180 — R^2 = 0.82
Patient #181 — R^2 = 0.936
Patient #182 — R^2 = 0.815
Patient #183 — R^2 = 0.77
Patient #184 — R^2 = 0.973
Patient #185 — R^2 = 0.894
Patient #186 — R^2 = 0.949
Patient #187 — R^2 = 0.867
Patient #188 — R^2 = 0.767
Patient #189 — R^2 = 0.996
Patient #190 — R^2 = 0.995
Patient #191 — R^2 = 0.115
Patient #192 — R^2 = 0.103

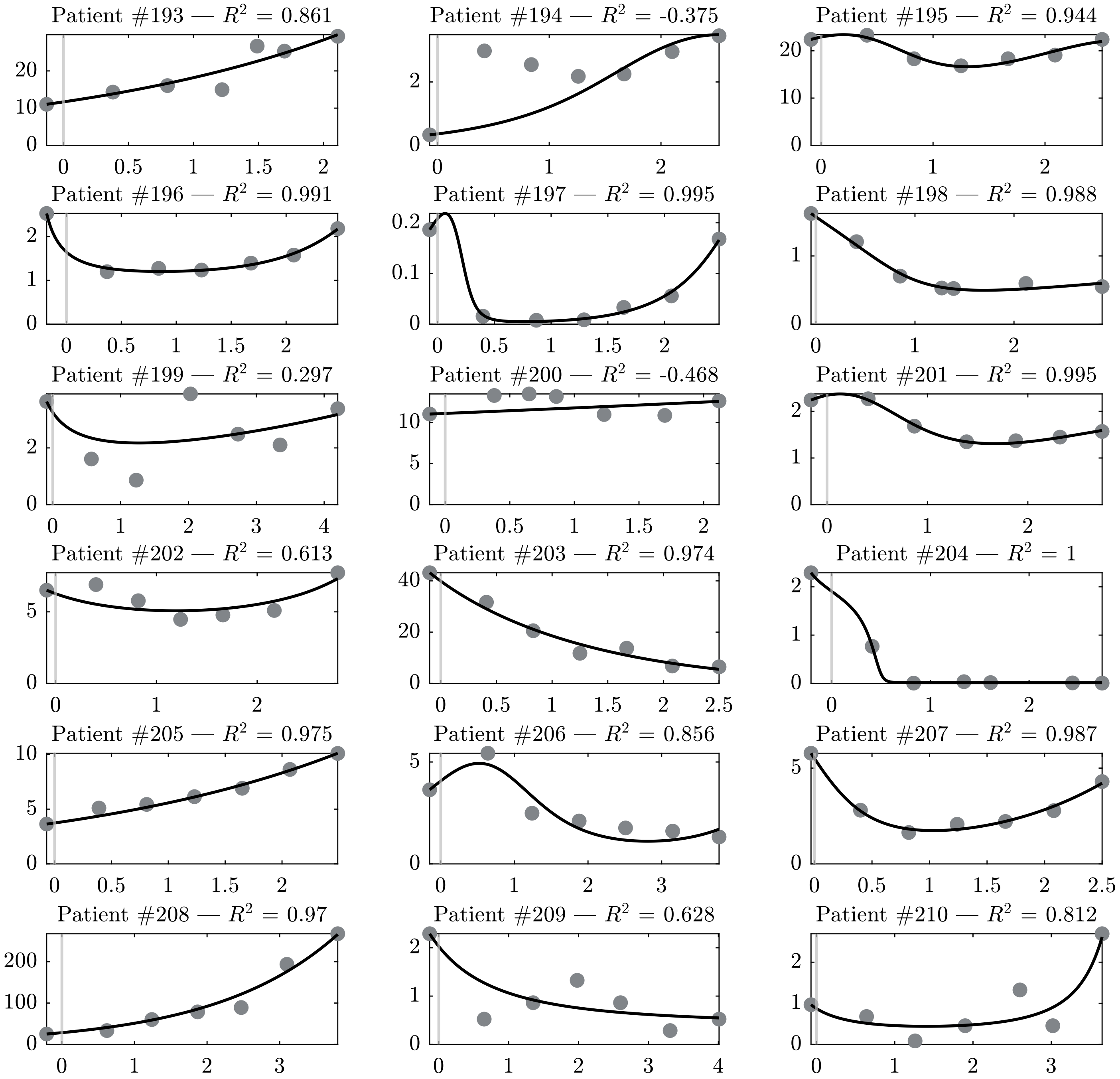
Patient #193 — R^2 = 0.861
0 10 20
0 0.5 1 1.5 2
Patient #194 — R^2 = -0.375
0 2
0 1 2
Patient #195 — R^2 = 0.944
0 10 20
0 1 2
Patient #196 — R^2 = 0.991
0 1 2
0 0.5 1 1.5 2
Patient #197 — R^2 = 0.995
0 0.1 0.2
0 0.5 1 1.5 2
Patient #198 — R^2 = 0.988
0 1
0 1 2
Patient #199 — R^2 = 0.297
0 2
0 1 2 3 4
Patient #200 — R^2 = -0.468
0 5 10
0 0.5 1 1.5 2
Patient #201 — R^2 = 0.995
0 1 2
0 1 2
Patient #202 — R^2 = 0.613
0 5
0 1 2
Patient #203 — R^2 = 0.974
0 20 40
0 0.5 1 1.5 2 2.5
Patient #204 — R^2 = 1
0 1 2
0 1 2
Patient #205 — R^2 = 0.975
0 5 10
0 0.5 1 1.5 2
Patient #206 — R^2 = 0.856
0 5
0 1 2 3
Patient #207 — R^2 = 0.987
0 5
0 0.5 1 1.5 2 2.5
Patient #208 — R^2 = 0.97
0 100 200
0 1 2 3
Patient #209 — R^2 = 0.628
0 1 2
0 1 2 3 4
Patient #210 — R^2 = 0.812
0 1 2
0 1 2 3

Model extrapolation results of all 210 patients. The solid-line curves and the points represent model results and measured data, respectively. The last two data points are not used for parameters estimation. Ordinates: normalized number of tumor cells. Abscissas: normalized treatment time, negative values indicate time before the start of treatment. The model is capable of forecasting tumor dynamics qualitatively and sometimes quantitatively.

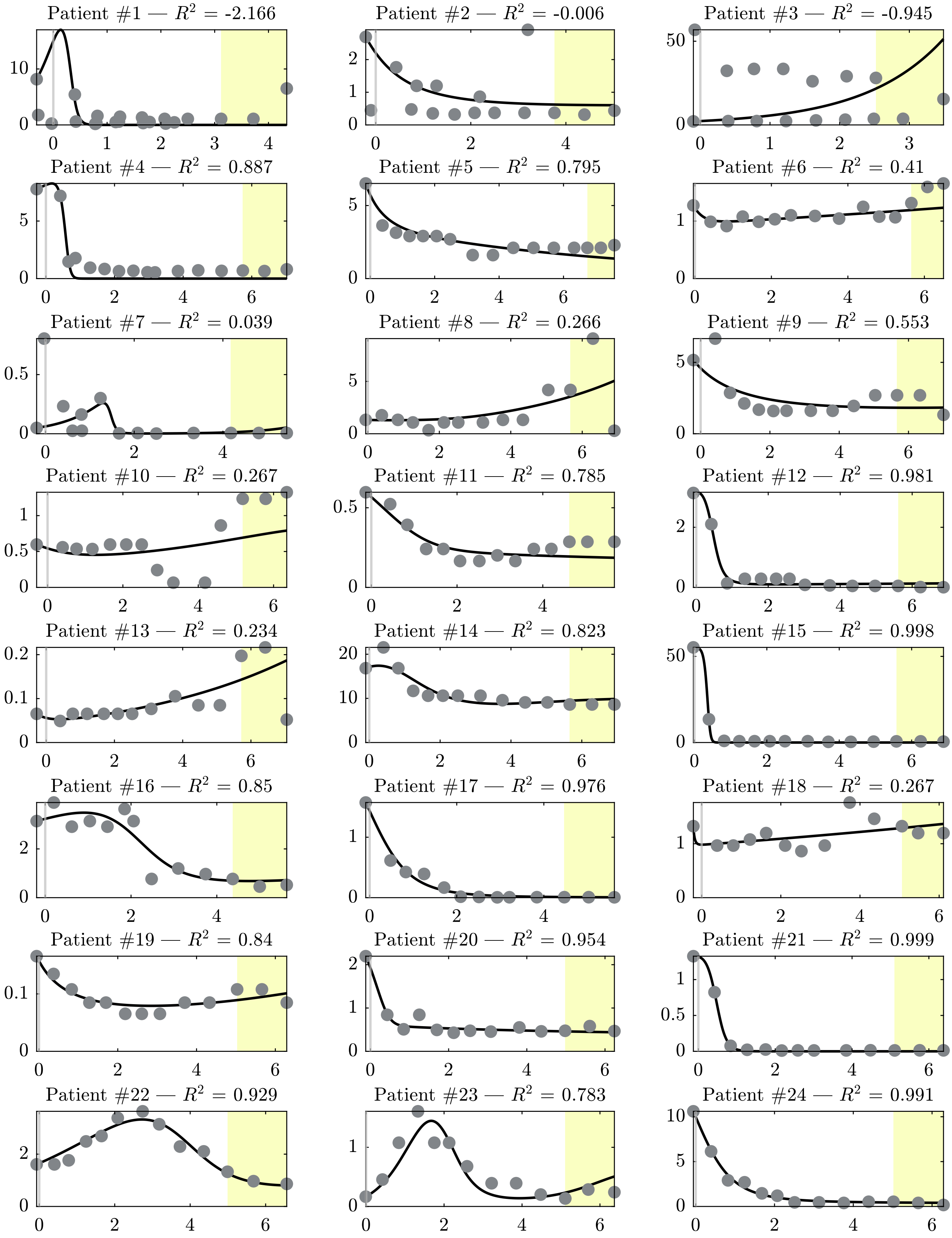
Patient #1 — R^2 = -2.166
Patient #2 — R^2 = -0.006
Patient #3 — R^2 = -0.945
Patient #4 — R^2 = 0.887
Patient #5 — R^2 = 0.795
Patient #6 — R^2 = 0.41
Patient #7 — R^2 = 0.039
Patient #8 — R^2 = 0.266
Patient #9 — R^2 = 0.553
Patient #10 — R^2 = 0.267
Patient #11 — R^2 = 0.785
Patient #12 — R^2 = 0.981
Patient #13 — R^2 = 0.234
Patient #14 — R^2 = 0.823
Patient #15 — R^2 = 0.998
Patient #16 — R^2 = 0.85
Patient #17 — R^2 = 0.976
Patient #18 — R^2 = 0.267
Patient #19 — R^2 = 0.84
Patient #20 — R^2 = 0.954
Patient #21 — R^2 = 0.999
Patient #22 — R^2 = 0.929
Patient #23 — R^2 = 0.783
Patient #24 — R^2 = 0.991

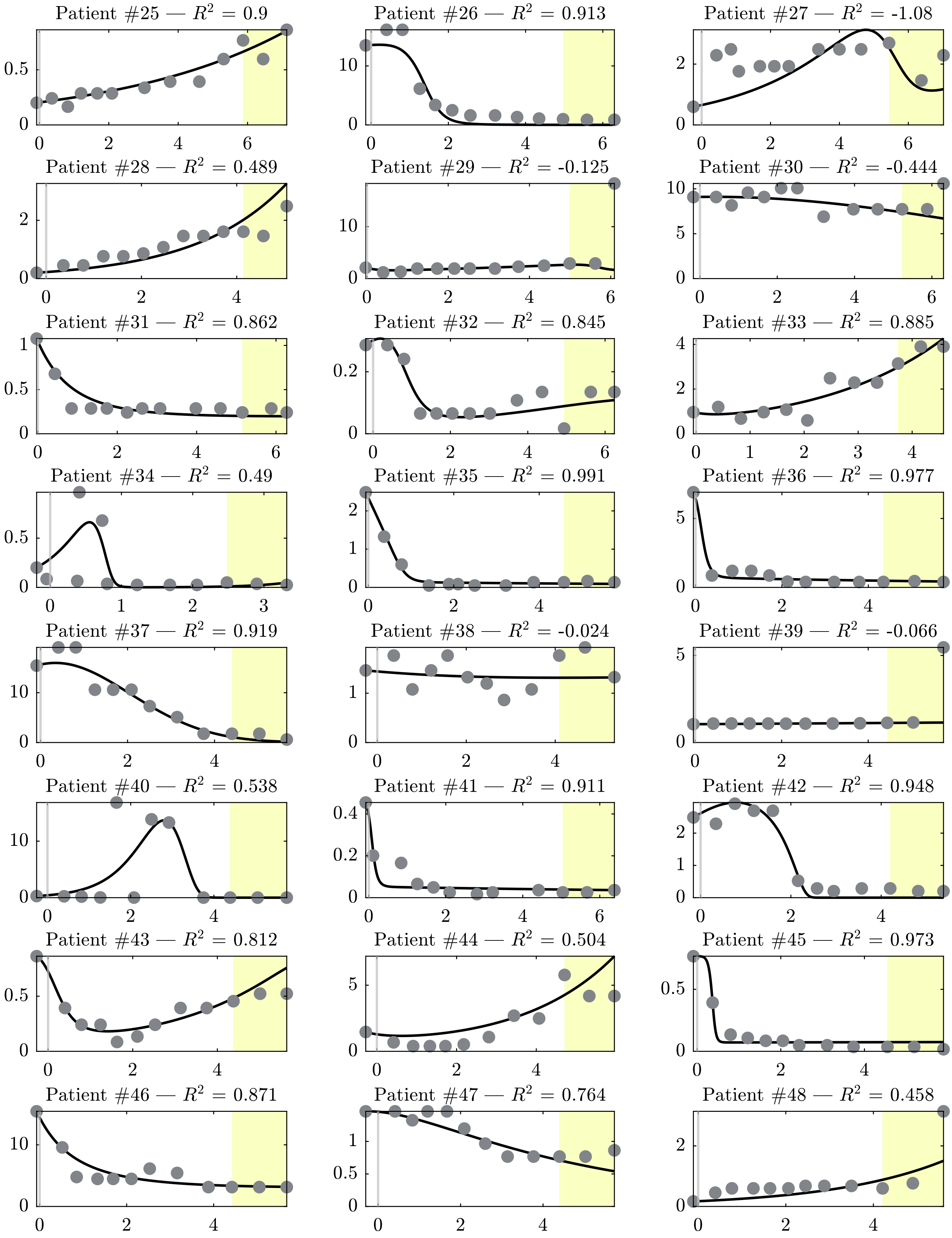

Patient #25 — R² = 0.9
Patient #26 — R² = 0.913
Patient #27 — R² = -1.08
Patient #28 — R² = 0.489
Patient #29 — R² = -0.125
Patient #30 — R² = -0.444
Patient #31 — R² = 0.862
Patient #32 — R² = 0.845
Patient #33 — R² = 0.885
Patient #34 — R² = 0.49
Patient #35 — R² = 0.991
Patient #36 — R² = 0.977
Patient #37 — R² = 0.919
Patient #38 — R² = -0.024
Patient #39 — R² = -0.066
Patient #40 — R² = 0.538
Patient #41 — R² = 0.911
Patient #42 — R² = 0.948
Patient #43 — R² = 0.812
Patient #44 — R² = 0.504
Patient #45 — R² = 0.973
Patient #46 — R² = 0.871
Patient #47 — R² = 0.764
Patient #48 — R² = 0.458

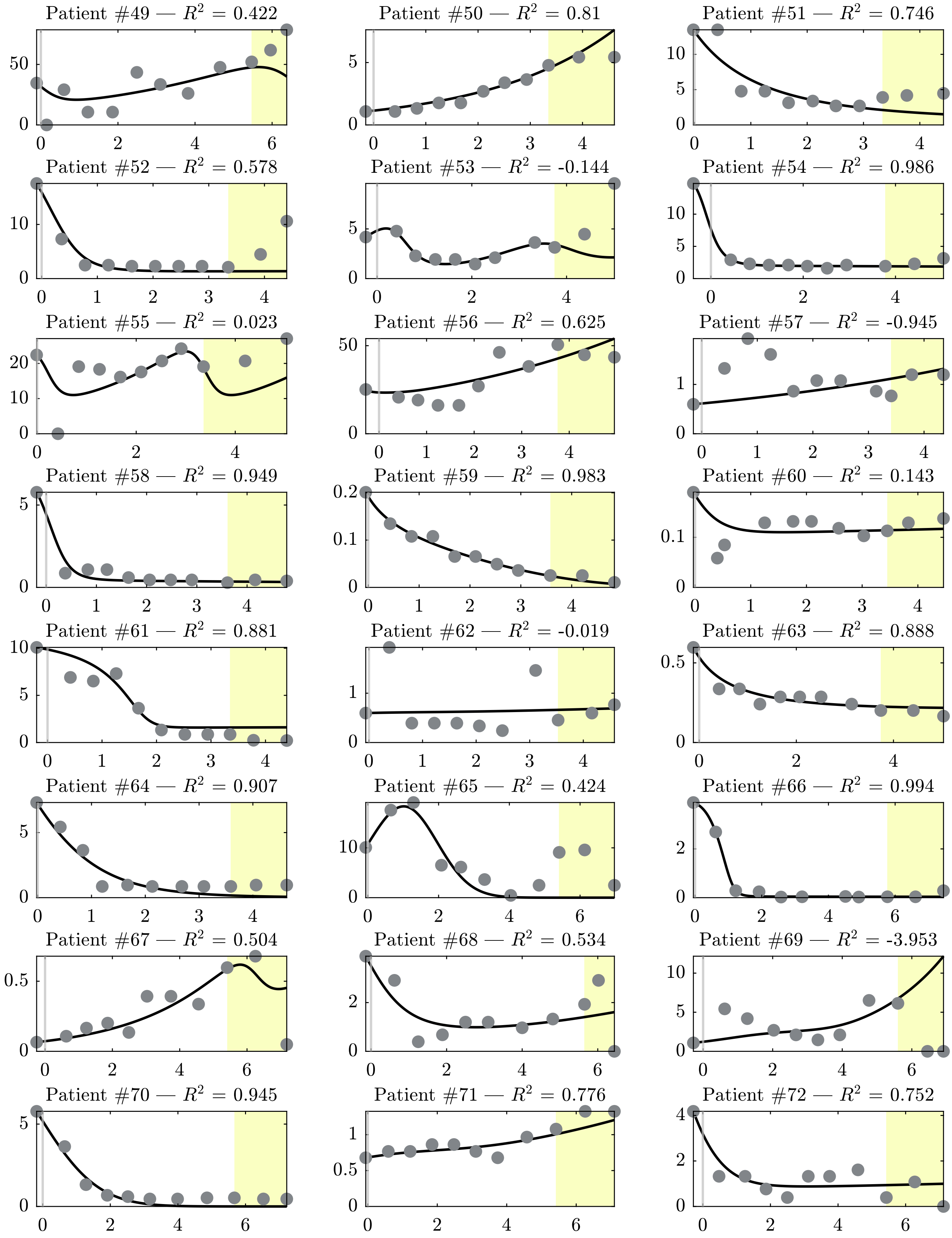
Patient #49 — R^2 = 0.422
Patient #50 — R^2 = 0.81
Patient #51 — R^2 = 0.746
Patient #52 — R^2 = 0.578
Patient #53 — R^2 = -0.144
Patient #54 — R^2 = 0.986
Patient #55 — R^2 = 0.023
Patient #56 — R^2 = 0.625
Patient #57 — R^2 = -0.945
Patient #58 — R^2 = 0.949
Patient #59 — R^2 = 0.983
Patient #60 — R^2 = 0.143
Patient #61 — R^2 = 0.881
Patient #62 — R^2 = -0.019
Patient #63 — R^2 = 0.888
Patient #64 — R^2 = 0.907
Patient #65 — R^2 = 0.424
Patient #66 — R^2 = 0.994
Patient #67 — R^2 = 0.504
Patient #68 — R^2 = 0.534
Patient #69 — R^2 = -3.953
Patient #70 — R^2 = 0.945
Patient #71 — R^2 = 0.776
Patient #72 — R^2 = 0.752

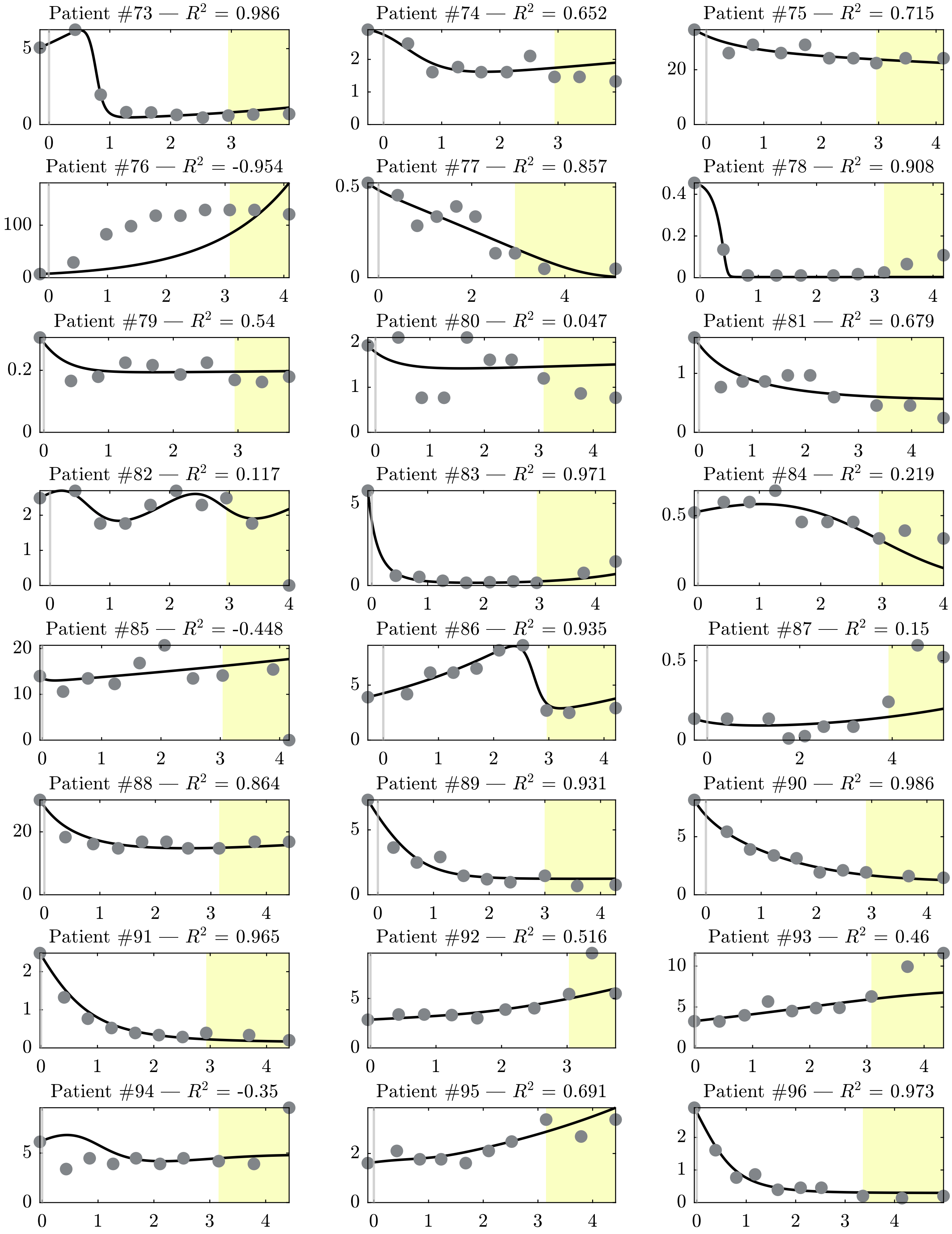
Patient #73 — R^2 = 0.986
Patient #74 — R^2 = 0.652
Patient #75 — R^2 = 0.715
Patient #76 — R^2 = -0.954
Patient #77 — R^2 = 0.857
Patient #78 — R^2 = 0.908
Patient #79 — R^2 = 0.54
Patient #80 — R^2 = 0.047
Patient #81 — R^2 = 0.679
Patient #82 — R^2 = 0.117
Patient #83 — R^2 = 0.971
Patient #84 — R^2 = 0.219
Patient #85 — R^2 = -0.448
Patient #86 — R^2 = 0.935
Patient #87 — R^2 = 0.15
Patient #88 — R^2 = 0.864
Patient #89 — R^2 = 0.931
Patient #90 — R^2 = 0.986
Patient #91 — R^2 = 0.965
Patient #92 — R^2 = 0.516
Patient #93 — R^2 = 0.46
Patient #94 — R^2 = -0.35
Patient #95 — R^2 = 0.691
Patient #96 — R^2 = 0.973

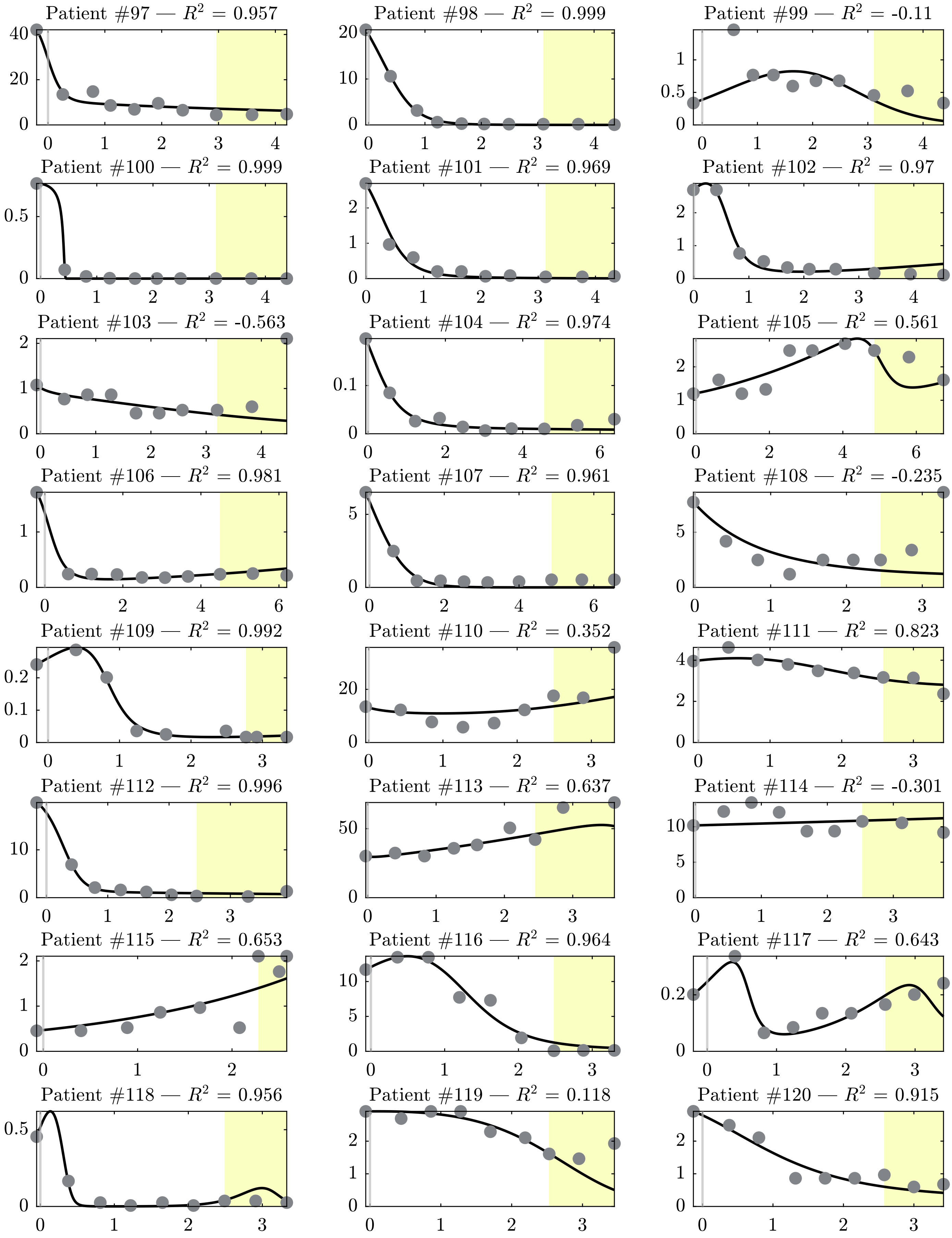
Patient #97 — R^2 = 0.957
Patient #98 — R^2 = 0.999
Patient #99 — R^2 = -0.11
Patient #100 — R^2 = 0.999
Patient #101 — R^2 = 0.969
Patient #102 — R^2 = 0.97
Patient #103 — R^2 = -0.563
Patient #104 — R^2 = 0.974
Patient #105 — R^2 = 0.561
Patient #106 — R^2 = 0.981
Patient #107 — R^2 = 0.961
Patient #108 — R^2 = -0.235
Patient #109 — R^2 = 0.992
Patient #110 — R^2 = 0.352
Patient #111 — R^2 = 0.823
Patient #112 — R^2 = 0.996
Patient #113 — R^2 = 0.637
Patient #114 — R^2 = -0.301
Patient #115 — R^2 = 0.653
Patient #116 — R^2 = 0.964
Patient #117 — R^2 = 0.643
Patient #118 — R^2 = 0.956
Patient #119 — R^2 = 0.118
Patient #120 — R^2 = 0.915

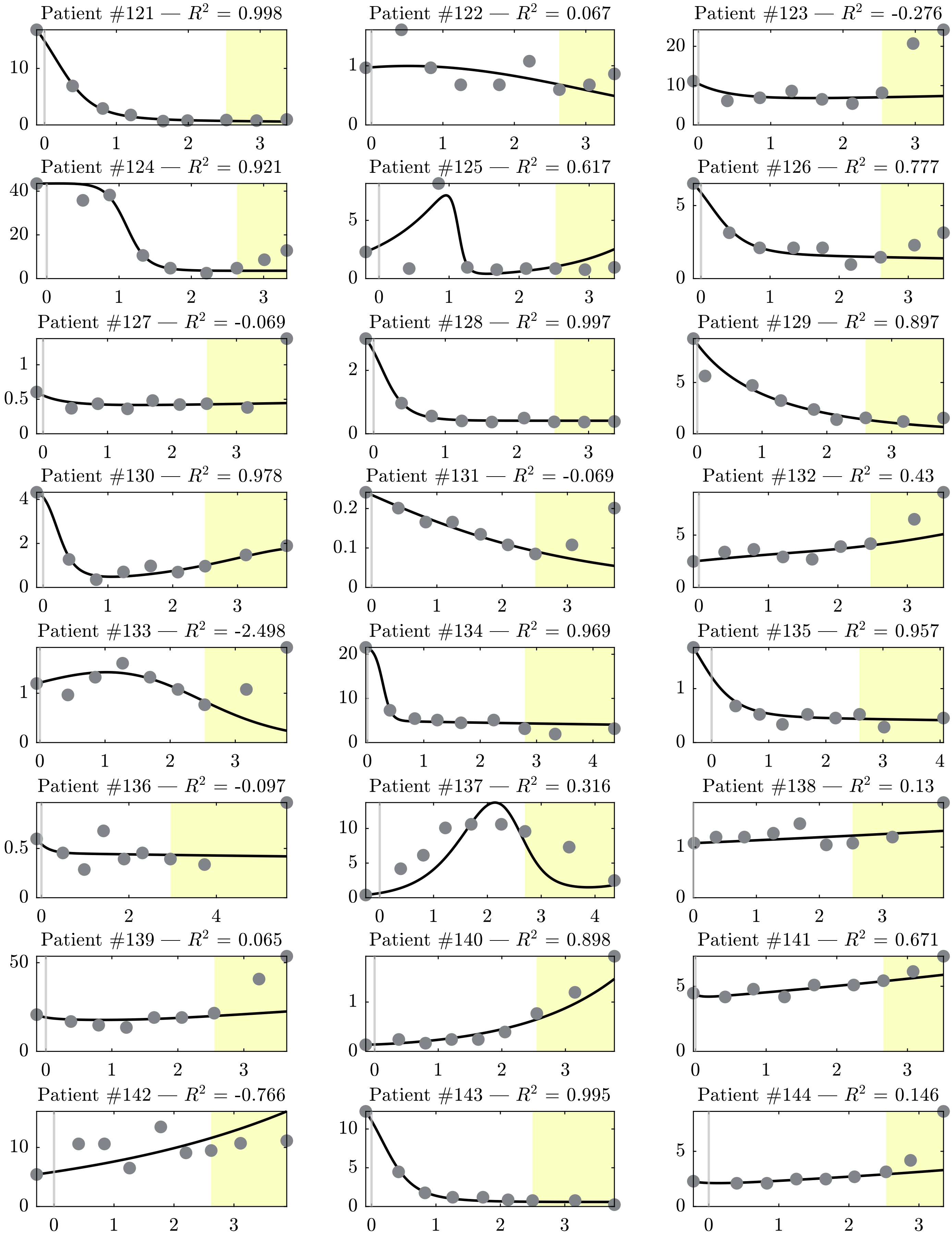

Patient #121 — R^2 = 0.998
Patient #122 — R^2 = 0.067
Patient #123 — R^2 = -0.276
Patient #124 — R^2 = 0.921
Patient #125 — R^2 = 0.617
Patient #126 — R^2 = 0.777
Patient #127 — R^2 = -0.069
Patient #128 — R^2 = 0.997
Patient #129 — R^2 = 0.897
Patient #130 — R^2 = 0.978
Patient #131 — R^2 = -0.069
Patient #132 — R^2 = 0.43
Patient #133 — R^2 = -2.498
Patient #134 — R^2 = 0.969
Patient #135 — R^2 = 0.957
Patient #136 — R^2 = -0.097
Patient #137 — R^2 = 0.316
Patient #138 — R^2 = 0.13
Patient #139 — R^2 = 0.065
Patient #140 — R^2 = 0.898
Patient #141 — R^2 = 0.671
Patient #142 — R^2 = -0.766
Patient #143 — R^2 = 0.995
Patient #144 — R^2 = 0.146

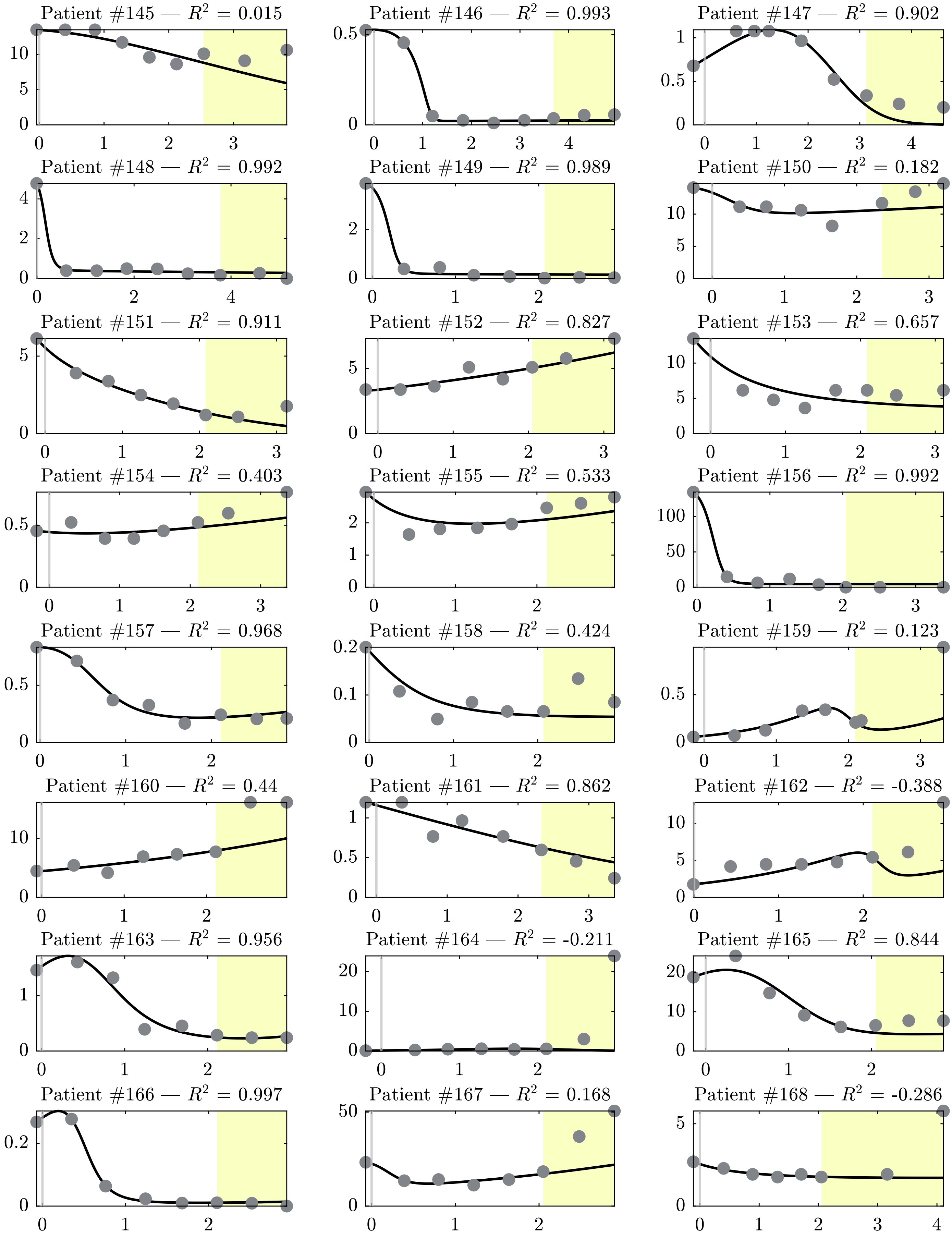
Patient #145 — R^2 = 0.015
Patient #146 — R^2 = 0.993
Patient #147 — R^2 = 0.902
Patient #148 — R^2 = 0.992
Patient #149 — R^2 = 0.989
Patient #150 — R^2 = 0.182
Patient #151 — R^2 = 0.911
Patient #152 — R^2 = 0.827
Patient #153 — R^2 = 0.657
Patient #154 — R^2 = 0.403
Patient #155 — R^2 = 0.533
Patient #156 — R^2 = 0.992
Patient #157 — R^2 = 0.968
Patient #158 — R^2 = 0.424
Patient #159 — R^2 = 0.123
Patient #160 — R^2 = 0.44
Patient #161 — R^2 = 0.862
Patient #162 — R^2 = -0.388
Patient #163 — R^2 = 0.956
Patient #164 — R^2 = -0.211
Patient #165 — R^2 = 0.844
Patient #166 — R^2 = 0.997
Patient #167 — R^2 = 0.168
Patient #168 — R^2 = -0.286

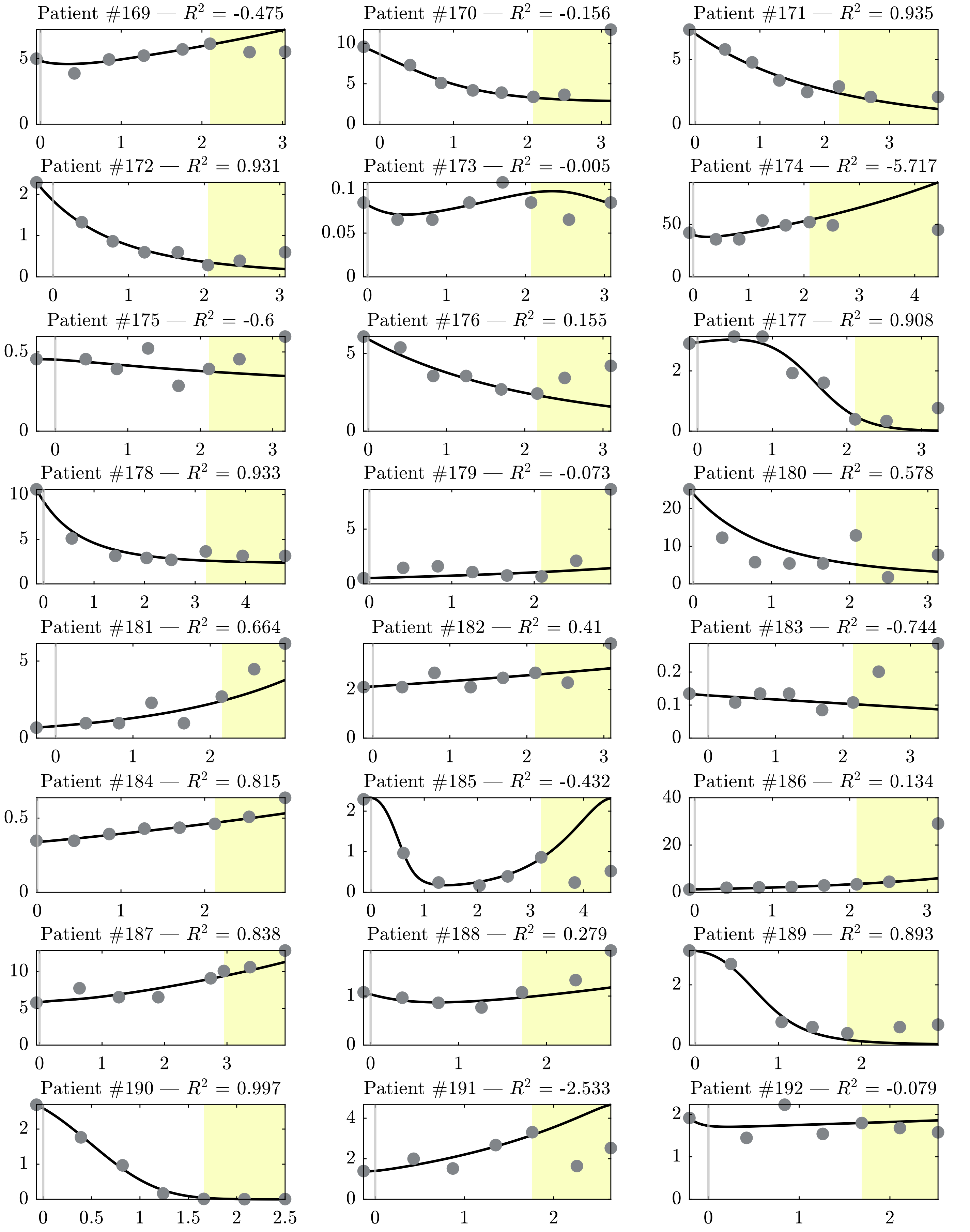

Patient #169 — R^2 = -0.475
Patient #170 — R^2 = -0.156
Patient #171 — R^2 = 0.935
Patient #172 — R^2 = 0.931
Patient #173 — R^2 = -0.005
Patient #174 — R^2 = -5.717
Patient #175 — R^2 = -0.6
Patient #176 — R^2 = 0.155
Patient #177 — R^2 = 0.908
Patient #178 — R^2 = 0.933
Patient #179 — R^2 = -0.073
Patient #180 — R^2 = 0.578
Patient #181 — R^2 = 0.664
Patient #182 — R^2 = 0.41
Patient #183 — R^2 = -0.744
Patient #184 — R^2 = 0.815
Patient #185 — R^2 = -0.432
Patient #186 — R^2 = 0.134
Patient #187 — R^2 = 0.838
Patient #188 — R^2 = 0.279
Patient #189 — R^2 = 0.893
Patient #190 — R^2 = 0.997
Patient #191 — R^2 = -2.533
Patient #192 — R^2 = -0.079

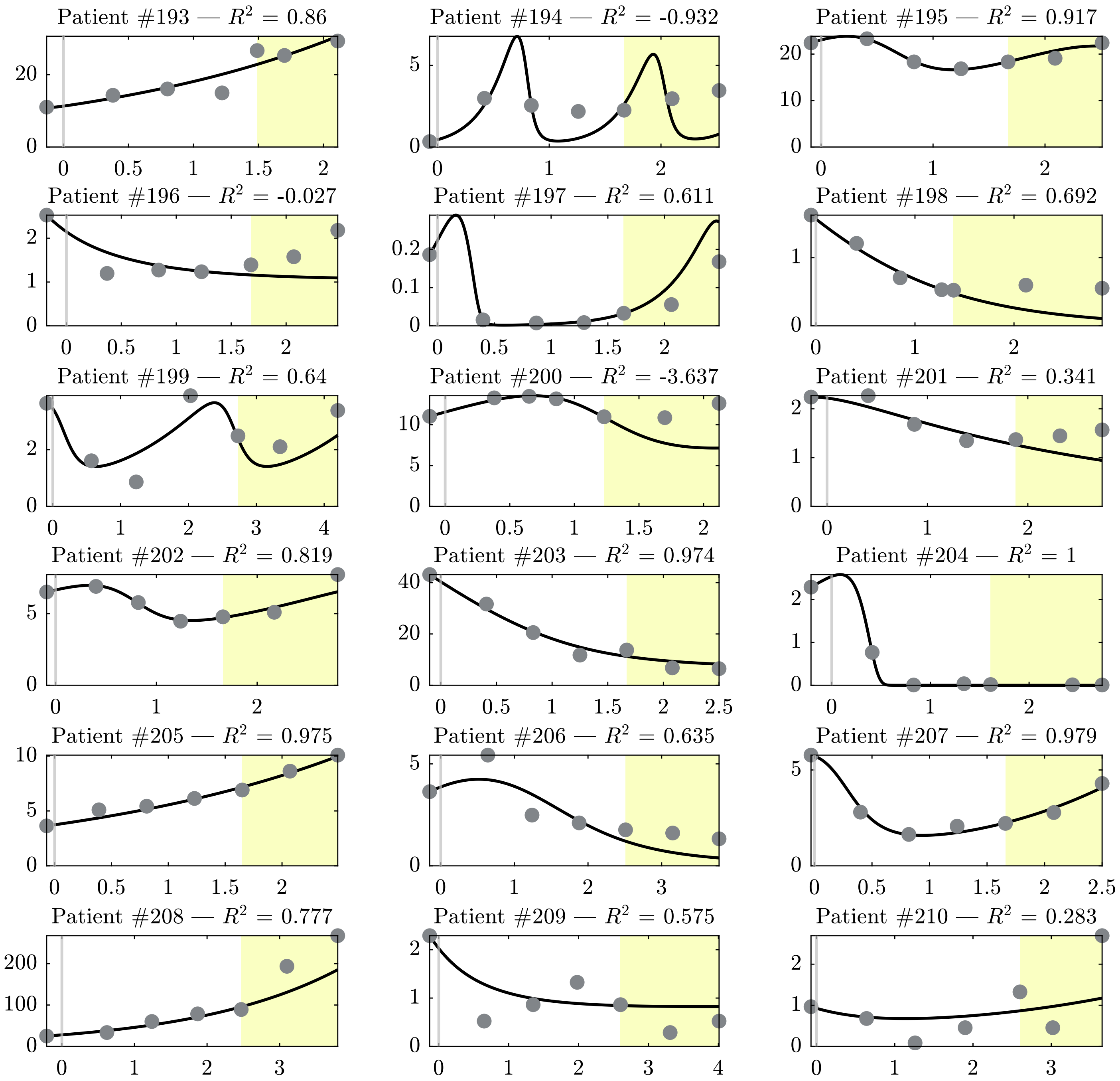
Patient #193 — R^2 = 0.86
Patient #194 — R^2 = -0.932
Patient #195 — R^2 = 0.917
Patient #196 — R^2 = -0.027
Patient #197 — R^2 = 0.611
Patient #198 — R^2 = 0.692
Patient #199 — R^2 = 0.64
Patient #200 — R^2 = -3.637
Patient #201 — R^2 = 0.341
Patient #202 — R^2 = 0.819
Patient #203 — R^2 = 0.974
Patient #204 — R^2 = 1
Patient #205 — R^2 = 0.975
Patient #206 — R^2 = 0.635
Patient #207 — R^2 = 0.979
Patient #208 — R^2 = 0.777
Patient #209 — R^2 = 0.575
Patient #210 — R^2 = 0.283

Identifiability analysis results of estimated values of model parameters of the selected patients. Ordinates: parameter values. Abscissas: non-dimensionalized model parameters. The points are the estimated values. The arrowheads are the maximum and minimum values found by the analysis. The results show that there are several combinations of parameter values at which the model can fit the experimental data.

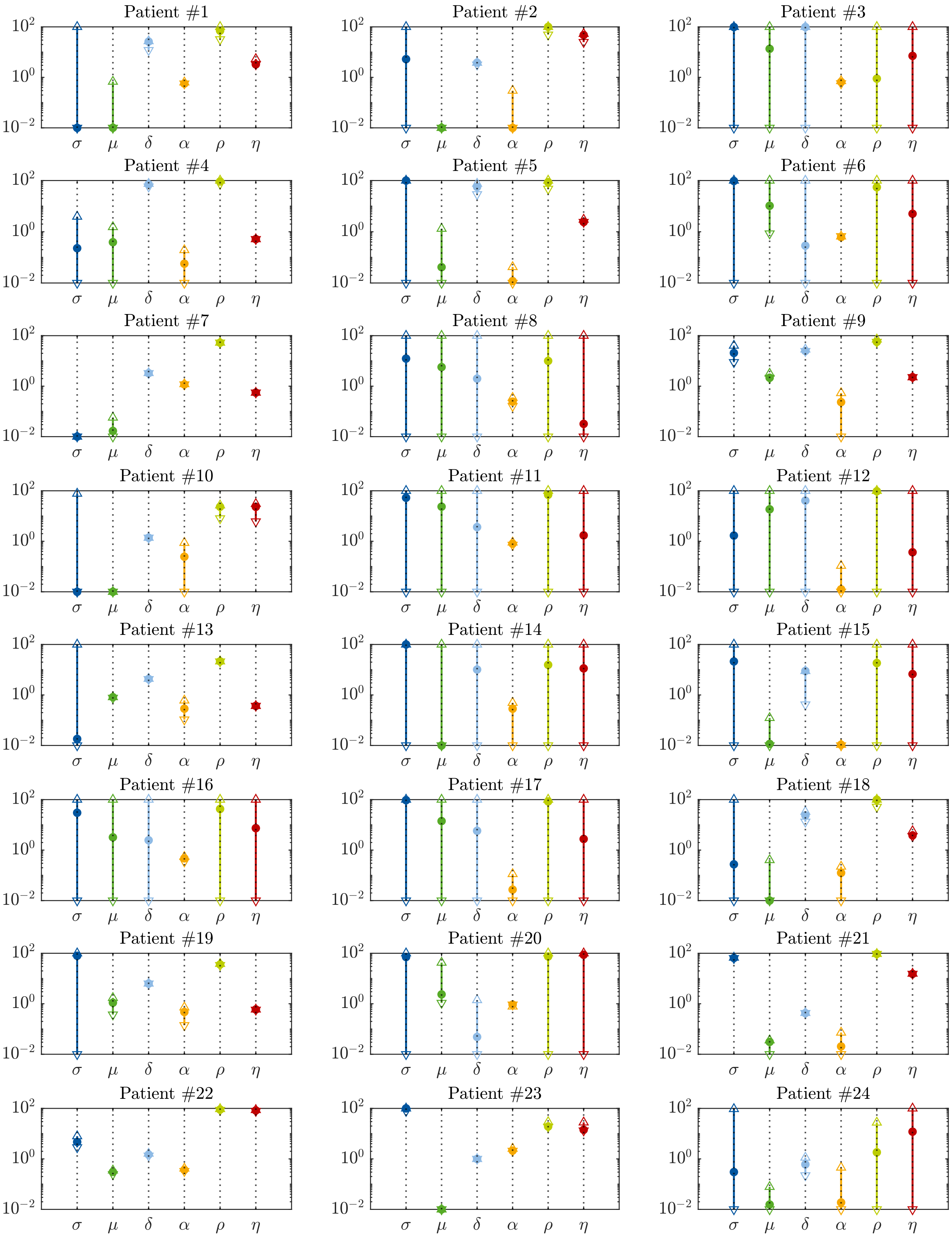

Patient #1
Patient #2
Patient #3
Patient #4
Patient #5
Patient #6
Patient #7
Patient #8
Patient #9
Patient #10
Patient #11
Patient #12
Patient #13
Patient #14
Patient #15
Patient #16
Patient #17
Patient #18
Patient #19
Patient #20
Patient #21
Patient #22
Patient #23
Patient #24
10^{2}
10^{0}
10^{-2}
σ μ δ α ρ η

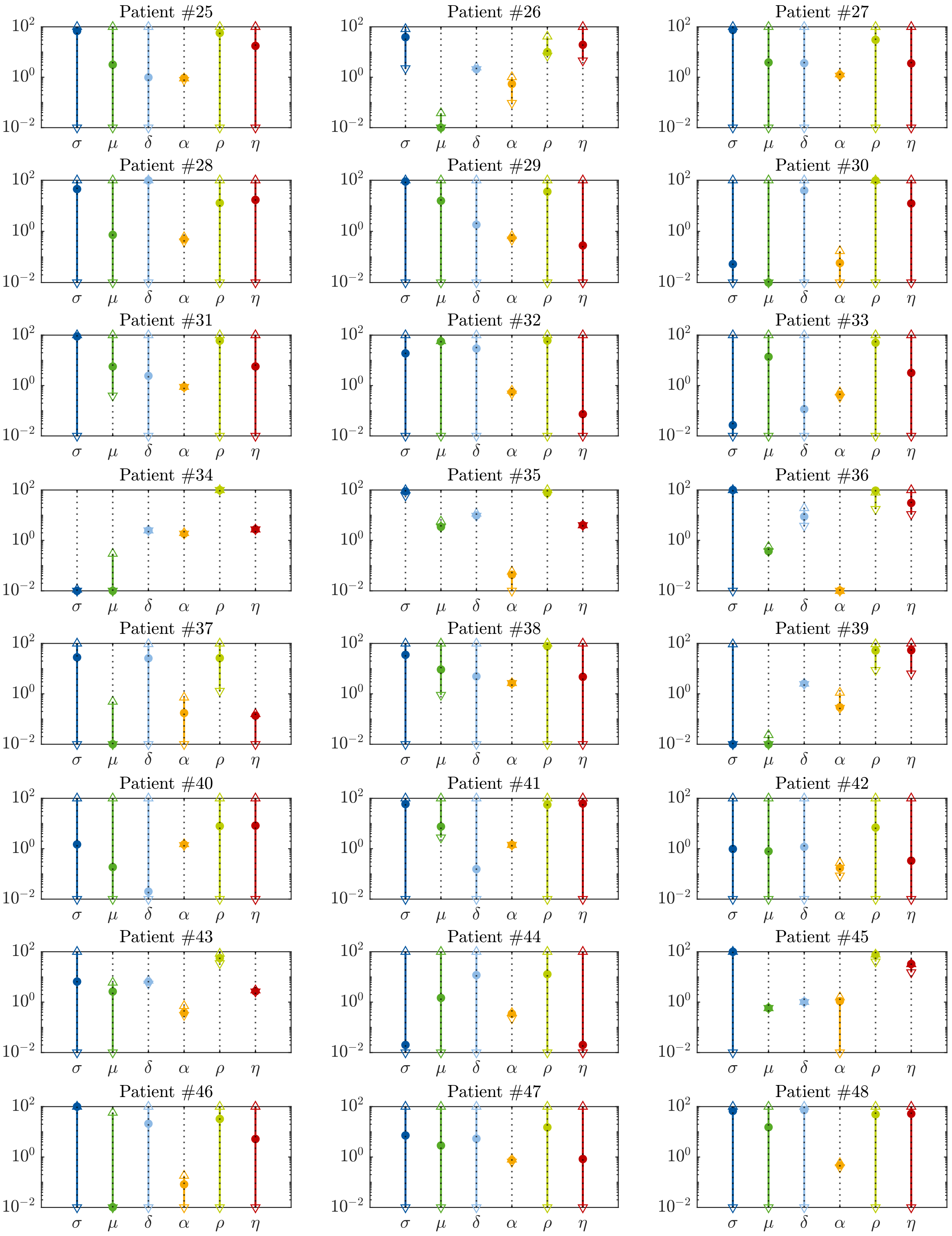
Patient #25
Patient #26
Patient #27
Patient #28
Patient #29
Patient #30
Patient #31
Patient #32
Patient #33
Patient #34
Patient #35
Patient #36
Patient #37
Patient #38
Patient #39
Patient #40
Patient #41
Patient #42
Patient #43
Patient #44
Patient #45
Patient #46
Patient #47
Patient #48
10^{2}
10^{0}
10^{-2}
σ μ δ α ρ η

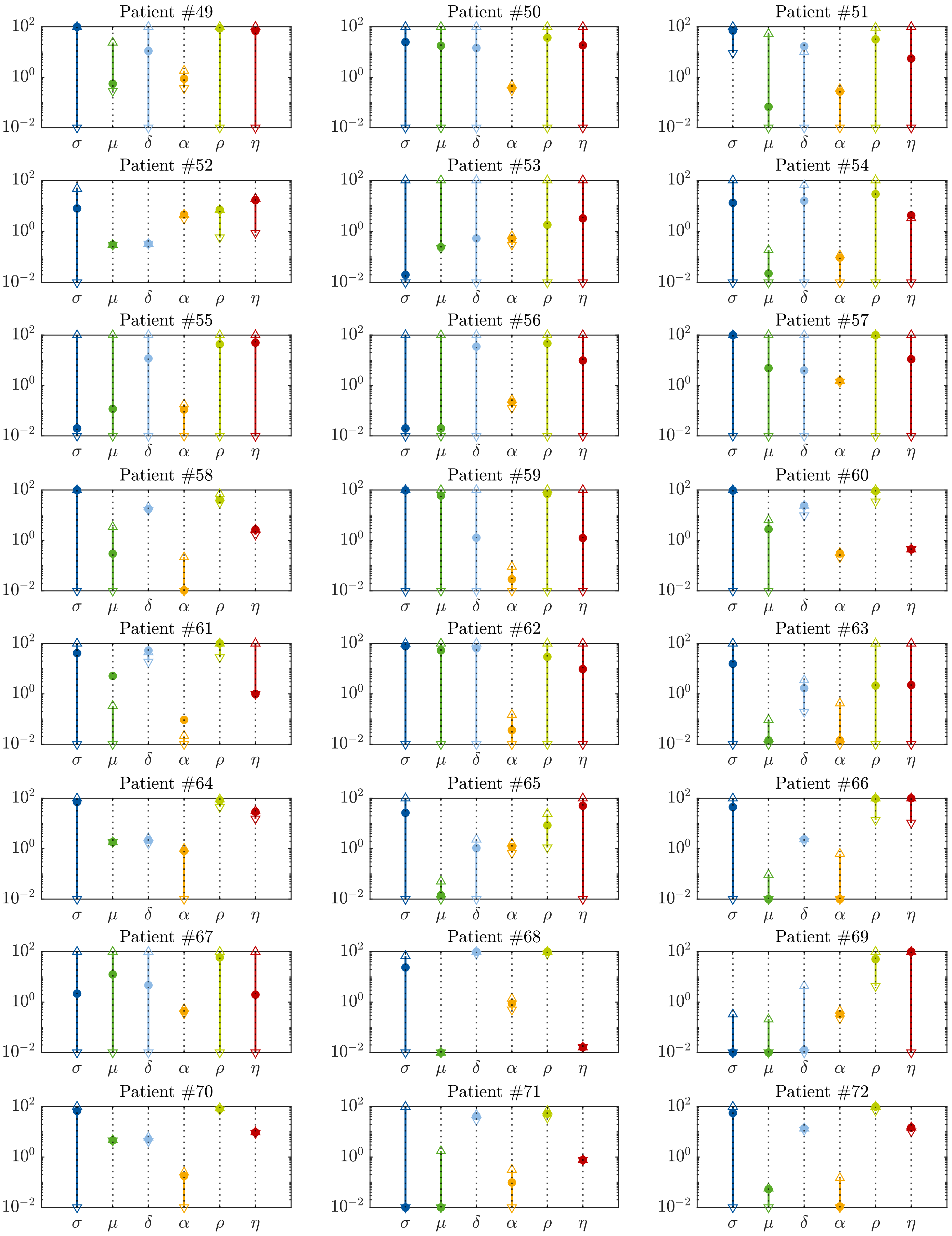

Patient #49
Patient #50
Patient #51
Patient #52
Patient #53
Patient #54
Patient #55
Patient #56
Patient #57
Patient #58
Patient #59
Patient #60
Patient #61
Patient #62
Patient #63
Patient #64
Patient #65
Patient #66
Patient #67
Patient #68
Patient #69
Patient #70
Patient #71
Patient #72
10^2
10^0
10^{-2}
σ μ δ α ρ η

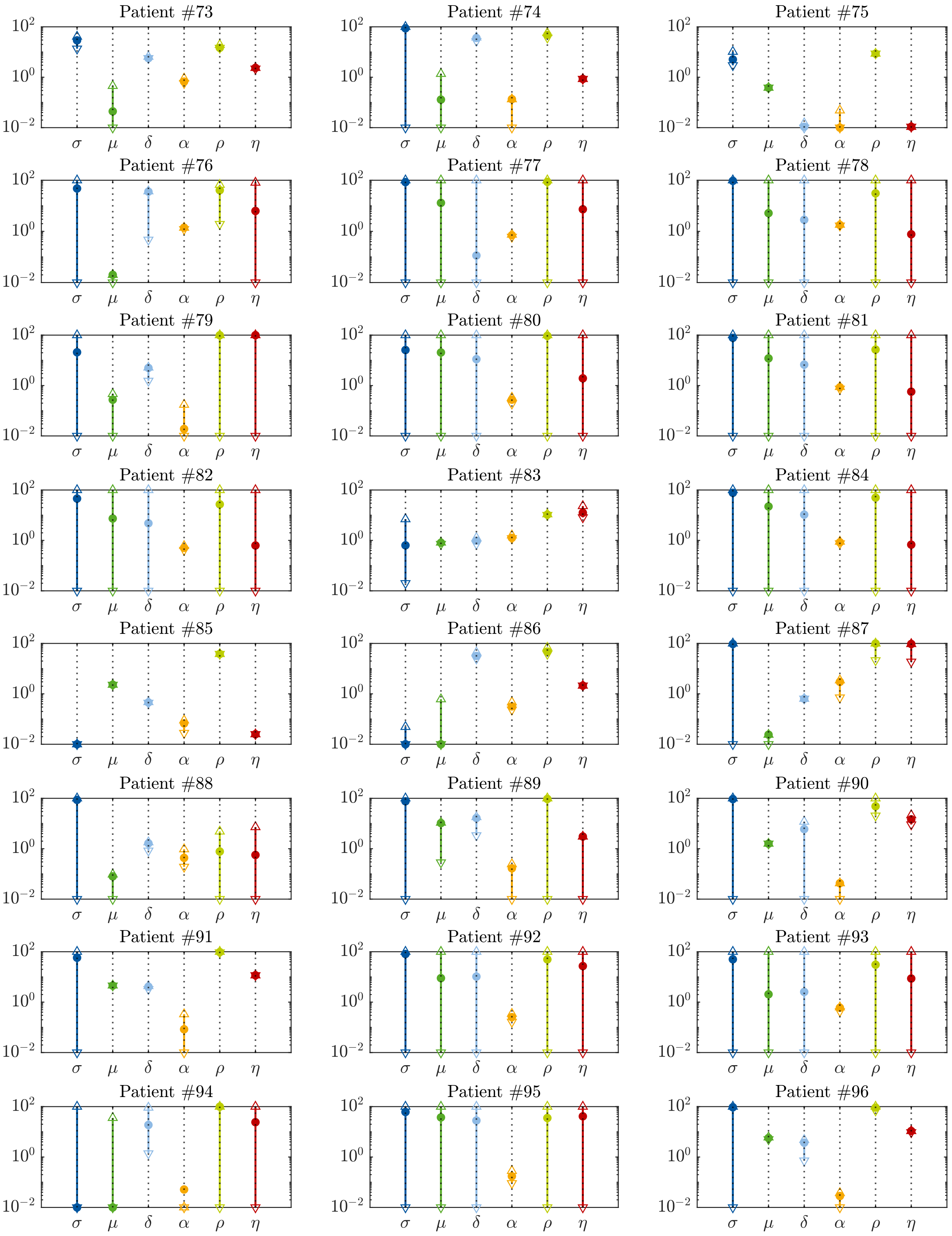

Patient #73
Patient #74
Patient #75
Patient #76
Patient #77
Patient #78
Patient #79
Patient #80
Patient #81
Patient #82
Patient #83
Patient #84
Patient #85
Patient #86
Patient #87
Patient #88
Patient #89
Patient #90
Patient #91
Patient #92
Patient #93
Patient #94
Patient #95
Patient #96
10^2
10^0
10^{-2}
σ μ δ α ρ η

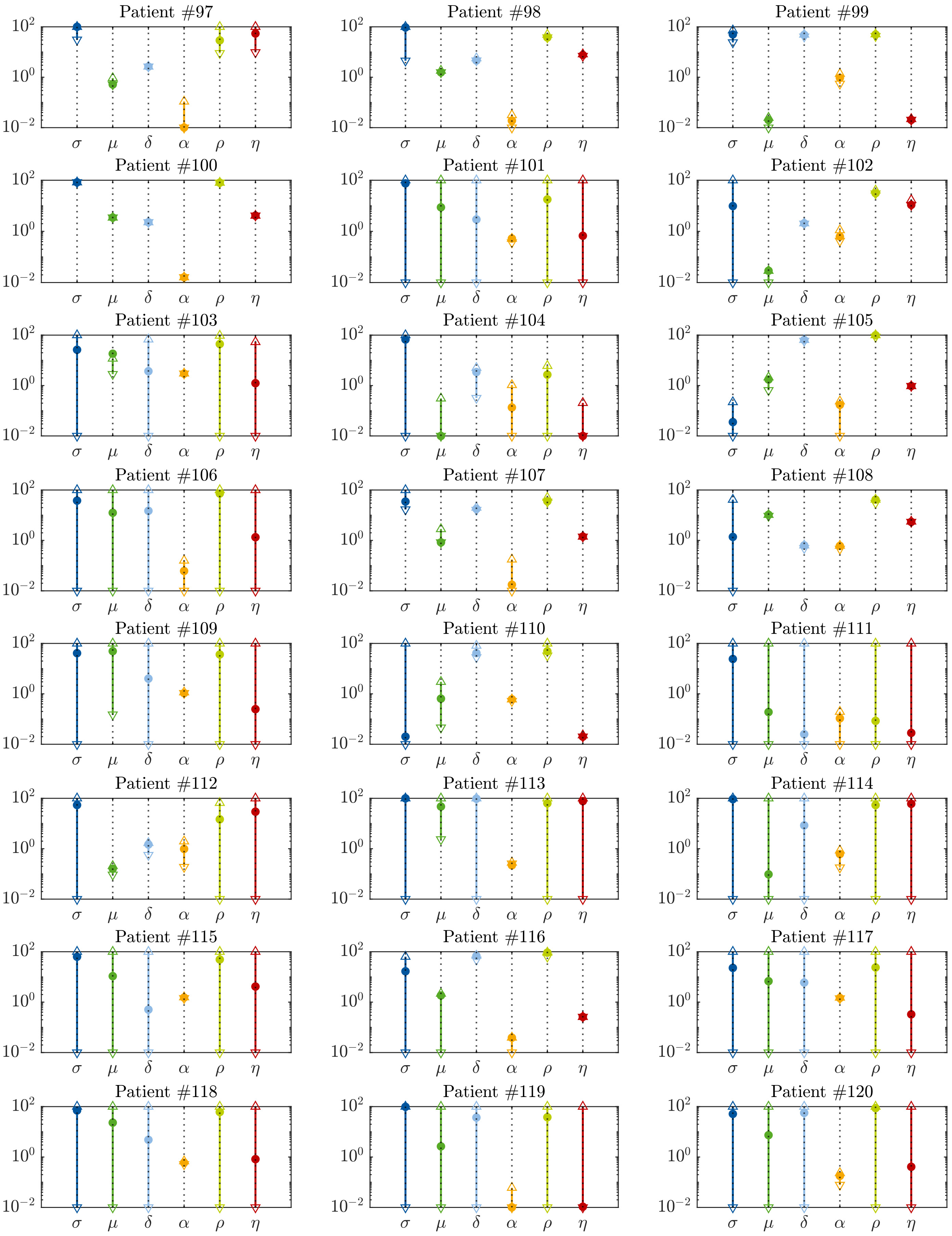
Patient #97
Patient #98
Patient #99
Patient #100
Patient #101
Patient #102
Patient #103
Patient #104
Patient #105
Patient #106
Patient #107
Patient #108
Patient #109
Patient #110
Patient #111
Patient #112
Patient #113
Patient #114
Patient #115
Patient #116
Patient #117
Patient #118
Patient #119
Patient #120
10^2
10^0
10^{-2}
σ μ δ α ρ η

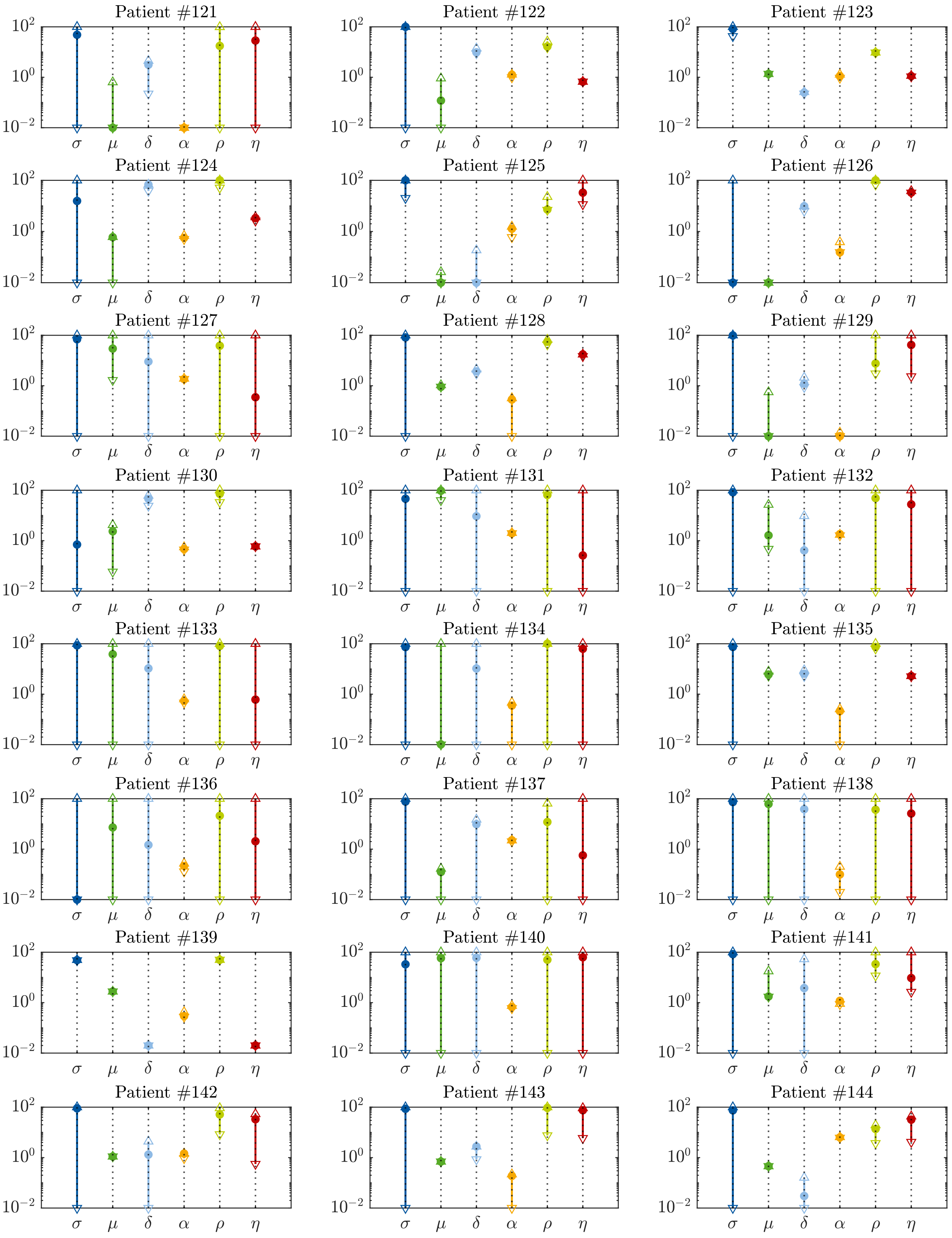

Patient #121
Patient #122
Patient #123
Patient #124
Patient #125
Patient #126
Patient #127
Patient #128
Patient #129
Patient #130
Patient #131
Patient #132
Patient #133
Patient #134
Patient #135
Patient #136
Patient #137
Patient #138
Patient #139
Patient #140
Patient #141
Patient #142
Patient #143
Patient #144
10^{2}
10^{0}
10^{-2}
σ μ δ α ρ η

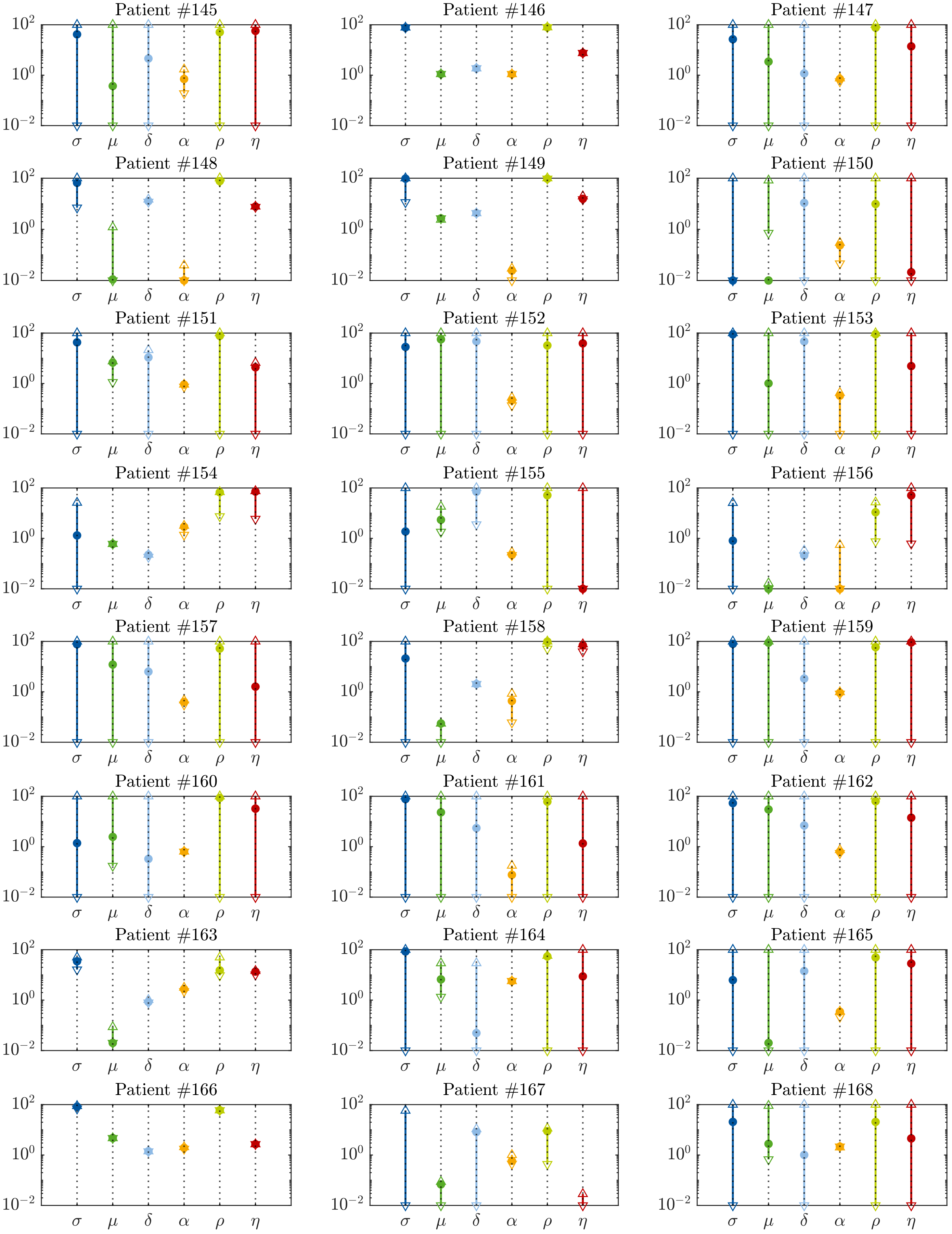
Patient #145
Patient #146
Patient #147
Patient #148
Patient #149
Patient #150
Patient #151
Patient #152
Patient #153
Patient #154
Patient #155
Patient #156
Patient #157
Patient #158
Patient #159
Patient #160
Patient #161
Patient #162
Patient #163
Patient #164
Patient #165
Patient #166
Patient #167
Patient #168
10^2
10^0
10^{-2}
σ μ δ α ρ η

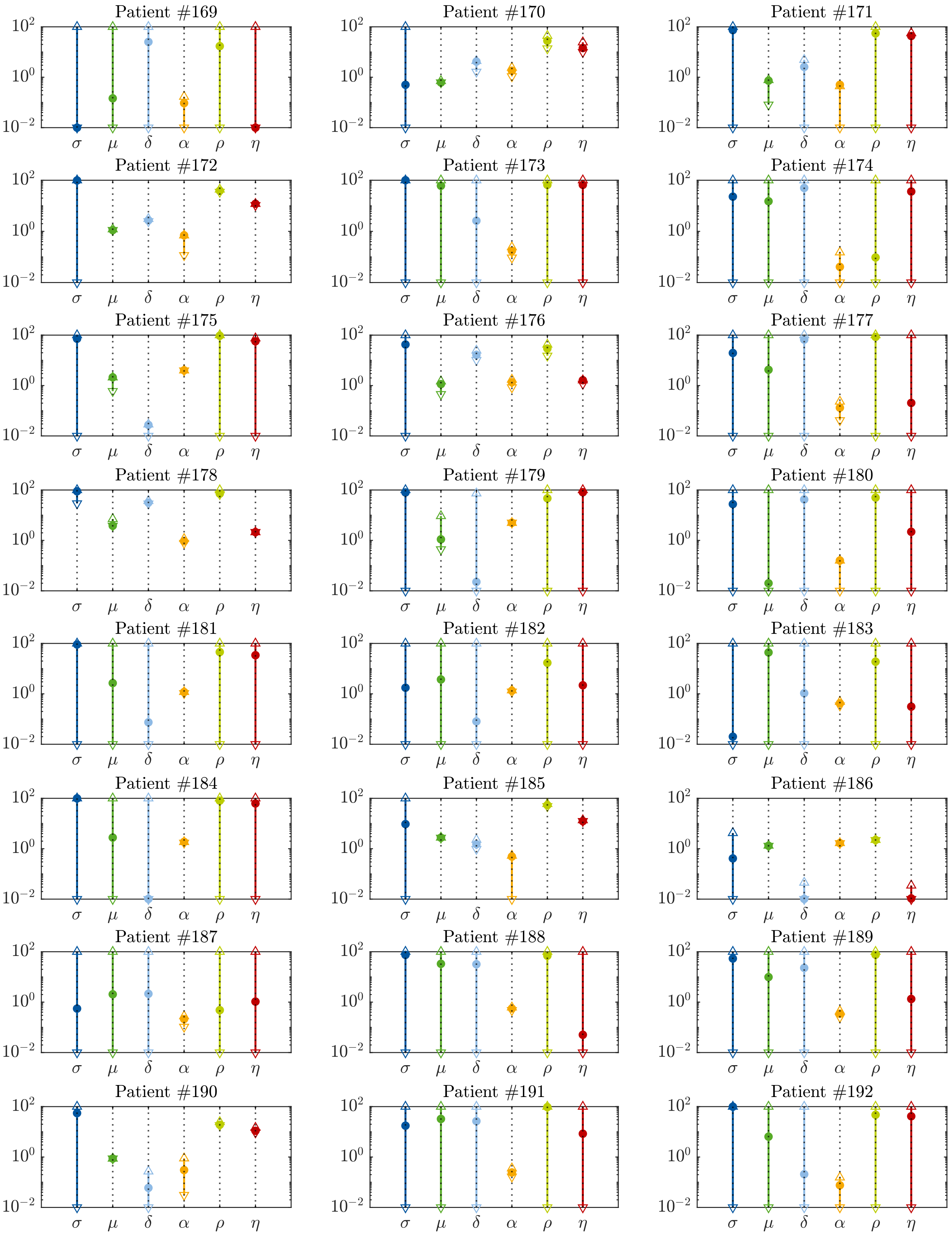

Patient #169
Patient #170
Patient #171
Patient #172
Patient #173
Patient #174
Patient #175
Patient #176
Patient #177
Patient #178
Patient #179
Patient #180
Patient #181
Patient #182
Patient #183
Patient #184
Patient #185
Patient #186
Patient #187
Patient #188
Patient #189
Patient #190
Patient #191
Patient #192
10^{2}
10^{0}
10^{-2}
σ μ δ α ρ η

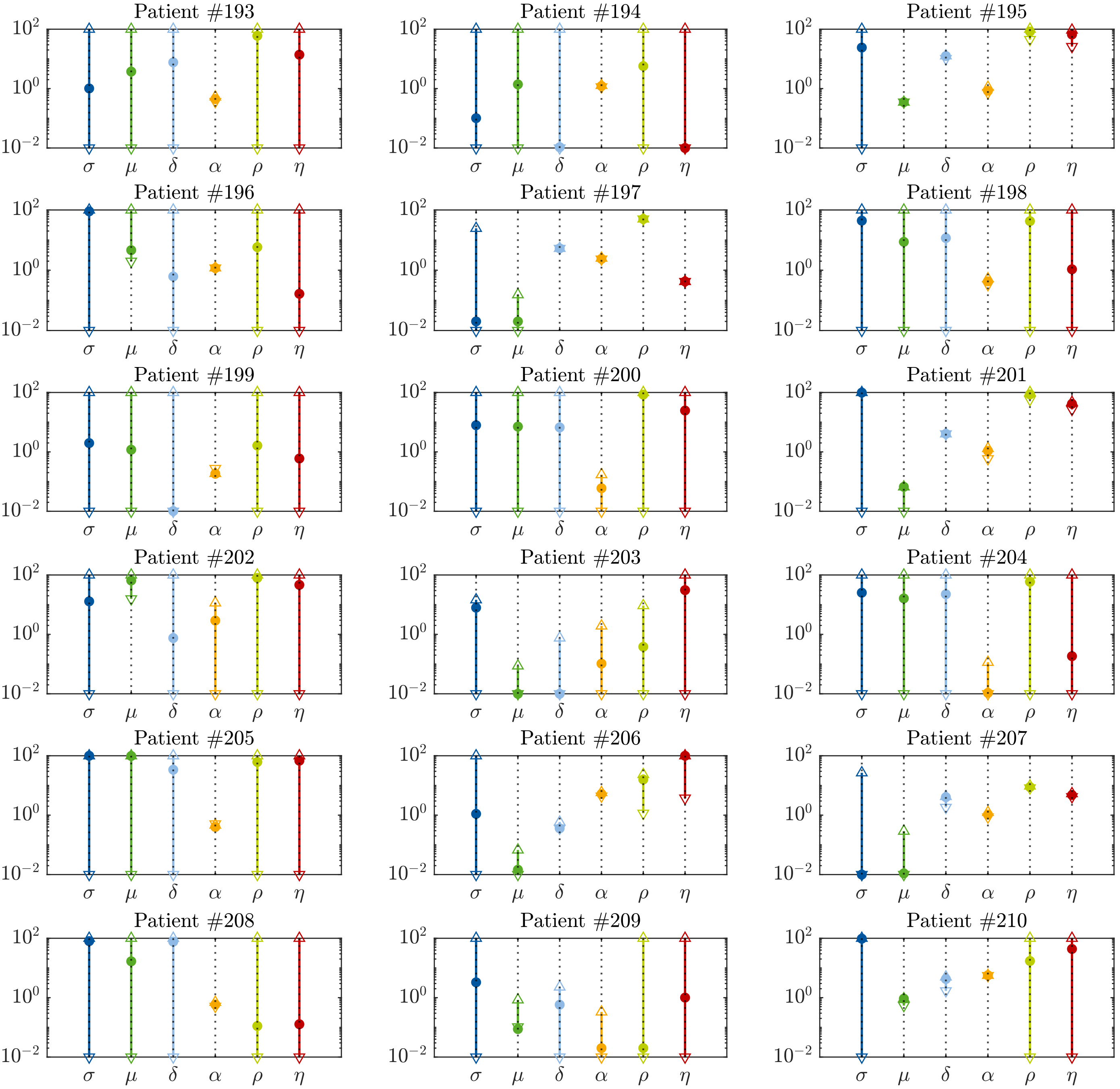

Patient #193
Patient #194
Patient #195
Patient #196
Patient #197
Patient #198
Patient #199
Patient #200
Patient #201
Patient #202
Patient #203
Patient #204
Patient #205
Patient #206
Patient #207
Patient #208
Patient #209
Patient #210
10^2
10^0
10^{-2}
σ μ δ α ρ η
